# Fusarisolins A–E, Polyketides from the Marine-Derived Fungus *Fusarium solani* H918

**DOI:** 10.3390/md17020125

**Published:** 2019-02-20

**Authors:** Siwen Niu, Xi-Xiang Tang, Zuowang Fan, Jin-Mei Xia, Chun-Lan Xie, Xian-Wen Yang

**Affiliations:** State Key Laboratory Breeding Base of Marine Genetic Resources, Key Laboratory of Marine Genetic Resources, Third Institute of Oceanography, Ministry of Natural Resources, 184 Daxue Road, Xiamen 361005, China; niusi123@126.com (S.N.); tangxixiang@tio.org.cn (X.-X.T.); fujianfanzuowang@126.com (Z.F.); xiajinmei@tio.org.cn (J.-M.X.); xiechunlanxx@163.com (C.-L.X.)

**Keywords:** ocean, microorganisms, β-lactones, HMG-CoA synthase, bioactivity

## Abstract

Five new (fusarisolins A–E, **1** to **5**) and three known (**6** to **8**) polyketides were isolated from the marine-derived fungus *Fusarium* *solani* H918, along with six known phenolics (**9** to **14**). Their structures were established by comprehensive spectroscopic data analyses, methoxyphenylacetic acid (MPA) method, chemical conversion, and by comparison with data reported in the literature. Compounds **1** and **2** are the first two naturally occurring 21 carbons polyketides featuring a rare β- and γ-lactone unit, respectively. All isolates (**1** to **14**) were evaluated for their inhibitory effects against tea pathogenic fungus *Pestalotiopsis theae* and 3-hydroxy-3-methylglutaryl coenzyme A (HMG-CoA) synthase gene expression. Compound **8** showed potent antifungal activity with an ED_50_ value of 55 μM, while **1**, **8**, **13**, and **14** significantly inhibited HMG-CoA synthase gene expression.

## 1. Introduction

Polyketides synthesized by polyketide synthases (PKS) are a large class of natural products with diverse structures and biological activities. The structural variations of polyketides are attributed to the post modification by tailoring enzymes and or a hybrid pathway, such as polyketide synthases−non-ribosomal peptide synthase (PKS-NRPS) [1]. β-Lactone featuring a strained four-membered oxygen heterocycle is a rare structural moiety in polyketides. To date, only 12 polyketides bearing β-lactone ring have been discovered in nature, including 1233A (also known as L-659,699 or F-244) [2,3,4], ebelactones A and B [5], obafluorin [6], belactosins A and C [7], lipstatin [8], salinosporamide A [9], vibralactone [10], vittatalactone [11], and cystargolides A and B [12]. Some of the polyketides have attracted wide attention owing to their potent pharmacological bioactivities [13,14,15]. For example, 1233A is a specific inhibitor of 3-hydroxy-3-methylglutaryl coenzyme A (HMG-CoA) synthase with an IC_50_ value of 0.12 µM [3]. Salinosporamide A has been shown to significantly inhibit proteasomal chymotrypsin-like proteolytic activity (IC_50_ = 1.3 nM) [9]. Lipstatin showed potent inhibitory effects against pancreatic lipase (IC_50_ = 0.14 µM) [8], and its derivative, tetrahydrolipstatin (orlistat), has been approved by the FDA to treat obesity [16].

**Figure 1:**
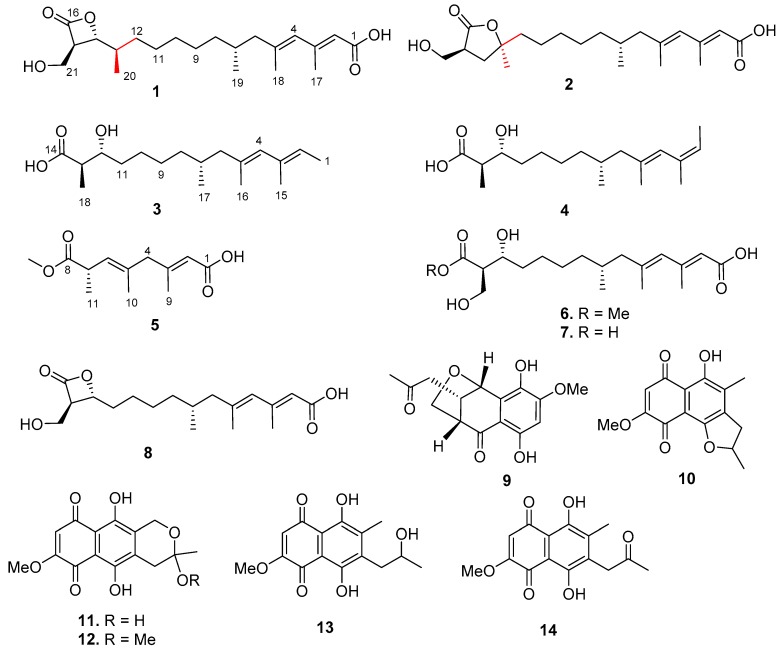
Chemical structures of **1** to **14** isolated from the *Fusarium solani* H918.

As part of our continuing discovery for structurally novel and biologically interesting secondary metabolites from marine microorganisms [17,18,19,20], the fungal strain *Fusarium solani* H918, isolated from mangrove sediments, was selected for a systematical chemical investigation due to its significant inhibitory activity against tea pathogenic fungus *Pestalotiopsis theae*. Extensive chromatographic separation of the EtOAc extract of the fermented cultures resulted in the isolation of eight polyketides (**1** to **8**) and six phenolic compounds (**9** to **14**) (Figure 1), of which compounds **1** and **2** are novel polyketides featuring a rare β- and γ-lactone ring, respectively. Herein, we report the isolation, structural elucidation, and bioactivities of these compounds.

## 2. Results and Discussion

**Figure 2:**
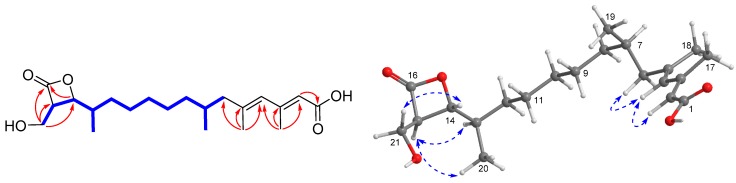
Selected COSY (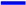), HMBC (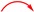), and NOESY (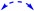) correlations of **1**.

Compound **1** was isolated as a colorless oil. The protonated molecular ion peak at *m*/*z* 367.2446 [M + H]^+^ (calcd for C_21_H_35_O_5_, 367.2426) in the high resolution electron spray ionization mass spectrum (HRESIMS) indicated its molecular formula as C_21_H_34_O_5_, requiring five degrees of unsaturation. The infrared absorption (IR) absorption at 1818 cm^−1^ indicated the presence of a β-lactone moiety. Its ^1^H NMR spectrum showed two signals as doublets (δ_H_ 0.87 and 1.04) and two as singlets (δ_H_ 1.82 and 2.20) assigned to the four methyl groups, one hydroxymethyl (δ_H_ 3.77, 3.91), two olefinic protons (δ_H_ 5.65 and 5.76), and one oxymethine (δ_H_ 4.31) (Table 1), while the ^13^C NMR spectrum exhibited 21 carbon resonance signals, including four methyls, seven methylenes, six methines (two olefinics), and four nonprotonated sp^2^ carbons (two carbonyls) (Table 2). Among them, four olefinic carbons (δ_C_ 119.1, 130.6, 142.2, and 155.2) for two double bonds and two ester carbonyl carbons (δ_C_ 171.0 and 172.1) accounted for four indices of hydrogen deficiency. The remaining one degree of unsaturation was due to the presence of a monocyclic ring in the molecule, which was consistent with the presence of a β-lactone ring as indicated by the IR spectrum. Analyses of the heteronuclear single quantum correlations (HSQC), ^1^H-^1^H correlated spectroscopy (COSY), and heteronuclear multiple bond correlations (HMBC) spectra of **1** determined a 1,2-dialkylated β-lactone skeleton, structurally close the coexisted antibiotics 1233A (**8**) [2]. The COSY cross-peaks of H-14 (δ_H_ 4.31)/H-15 (δ_H_ 3.53)/H_2_-21 (δ_H_ 3.91, 3.77) and the HMBC interactions from H_2_-21 to C-14 (δ_C_ 80.2), C-15 (δ_C_ 58.2), and C-16 (δ_C_ 172.1) and from H_3_-18 (δ_H_ 1.04) to C-12 (δ_C_ 32.4)/C-13 (δ_C_ 38.1)/C-14 revealed a hydroxymethyl and a alkyl chain to be attached to C-15 and C-14 positions, respectively (Figure 2). The alkyl chain was expanded from C-12 to C-1 (δ_C_ 171.0) as evidenced by the contiguous COSY cross-peaks from H_2_-6 (δ_H_ 2.13, 1.88) to H_2_-12 (δ_H_ 1.44, 1.18), H-7 (δ_H_ 1.70)/H_3_-19 (δ_H_ 0.87), and H-13 (δ_H_ 1.82)/H_3_-20 (δ_H_ 1.04), together with the HMBC relationships originating from H-2 (δ_H_ 5.65), H_3_-17 (δ_H_ 2.20), and H_3_-18 (δ_H_ 1.82) to the corresponding carbons as shown in Figure 2. On the basis of the above data, the alkyl side chain with 13 carbons bearing four methyl groups was elucidated as a 3,5,7-trimethyl-tetradeca-2,4-dienoic acid. Therefore, the gross structure of **1** was determined as a novel 21 carbons polyketide featuring a rare β-lactone moiety.

The geometries of double bonds at Δ^2^ and Δ^4^ were established as 2*E* and 4*E* configurations by the nuclear overhauser effect (NOESY) correlations from H-4 (δ_H_ 5.76) to H-2 and H_2_-6 (Figure 2). Additional NOESY relationships from H_2_-21 to H-14, in association with the similar coupling constants of **1** (*J*_H-14/H-15_ = 4.2 Hz), 1233A (*J* = 4.2 Hz), and vittatalactone (*J* = 4.0 Hz) indicated the trans-relationship of H-14 and H-15 in the β-lactone ring [11,21]. The same orientation of H-13 and H-15 was presumed by the NOESY cross-peaks from H-15 to H-13 and H_3_-20 (Figure 2). Absolute configuration of C-15 was determined by application of the MPA method used for establishing absolute configuration and optical purity of primary alcohols with chiral center at C-2 position [22,23]. The chemical shift difference between two methylene protons of H_2_-21 in (*R*)-MPA ester (**1a**) (Δδ_H_ 0.08) is larger than that of (*S*)-MPA ester (**1b**) (Δδ_H_ 0.05), revealing 15*R* configuration. Additionally, the absolute configuration of C-7 at linear side chain was presumed to be the same as that of **8** according to the biogenetic considerations, which was based on the fact that the isolate **8** was established as 1233A by comparison of their NMR data and specific rotation values between **8** ($[\alpha {]}_{D}^{25}$ 25.6, CHCl_3_) and 1233A ($[\alpha {]}_{D}^{25}$ 27.5, CHCl_3_) [2,24]. Therefore, the structure of **1** was then established as a novel (2*E*,4*E*,7*R*,13*R*)-13-((2*R*,3*R*)-3-(hydroxymethyl)-4-oxooxetan-2-yl)-3,5,7-trimethyltetradeca-2,4-dienoic acid, and given the name, fusarisolin A.

**Figure 3:**
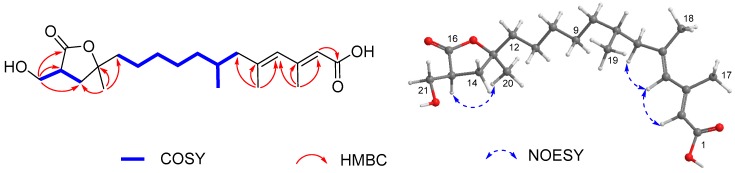
Key COSY, HMBC, and NOESY correlations of **2**.

Compound **2** exhibited the same molecular formula as that of **1** according to its HRESIMS spectrum. Interestingly, it also showed nearly identical ^13^C NMR data to those of **1** revealing a structurally similar analogue. The difference was attributed to the methine of C-13 and oxymethine of C-14 of **1** being replaced by an oxygenated nonprotonated sp^3^ carbon (δ_C_ 86.5) and methylene (δ_C_ 36.5), respectively, suggesting the presence of a γ-lactone instead of a β-lactone unit in **2**. This assumption was evidenced by the HMBC correlations from H_3_-20 (δ_H_ 1.38) to C-12 (δ_C_ 42.6)/C-13(δ_C_ 86.5)/C-14 (δ_C_ 36.5) and from H_2_-21 (δ_H_ 3.87, 3.72) to C-14/C-15 (δ_C_ 44.6)/C-16 (δ_C_ 179.5), and the COSY correlations of H_2_-14 (δ_H_ 2.16)/H-15 (δ_H_ 3.02)/H_2_-21 (Figure 3), in addition to the same molecular formula as well as the comparisons of the corresponding NMR data of C-13/C-14/C-15/C-16/C-21 (δ_C_ 61.0) of **2** with those of the structurally related compound 3-hydroxymethyl-5,5-dimethyl-dihydro-2(3*H*)furanone (corresponding to δ_C_ 83.2, 37.1, 43.3, 177.8, and 60.9, respectively) [25]. In the NOESY spectrum, H-15 was correlated to H_3_-20 indicating same orientation of these protons (Figure 3). Additionally, the configurations of the conjugated double bonds at Δ^2^ and Δ^4^ were determined to be same as those of **1** on the basis of the NOESY cross-peaks from H-4 (δ_H_ 5.76) to H-2 (δ_H_ 5.65) and H_2_-6 (δ_H_ 2.14, 1.89) (Figure 3). The absolute configuration of **2** was presumably assigned as 7*R*, 13*S*, and 15*S* on the basis of the biogenetic considerations between **2** and **1**. Considering chemical rearrangement of a β-lactone (similar to compound **1**) to a γ-lactone (similar to compound **2**) catalyzed by EtMgBr [26], structure of **2** might be derived from **1** after chemical rearrangement. Therefore, the novel structure of **2** was elucidated to be (2*E*,4*E*,7*R*)-12-((2*S*,4*S*)-4-(hydroxymethyl)-2-methyl-5-oxotetrahydrofuran-2-yl)-3,5,7-trimethyldodeca-2,4-dienoic acid, and named, fusarisolin B.

It is noteworthy that fusarisolins A (**1**) and B (**2**) are the first examples of 21-carbons polyketides bearing a rare β- and γ-lactone moiety, respectively.

Compound **3** exhibited the molecular formula of C_18_H_32_O_3_ as determined by negative HRESIMS spectrum (*m*/*z* 295.2256, [M − H]^−^). The ^13^C NMR spectrum showed 18 carbon resonance signals including five methyls, five methylenes, five methines (two olefinics), and three nonprotonated sp^2^ carbons (two olefinics and one carbonyl), which were similar to those of 12-*epi*-1233B (**7**) [2]. However, a close comparison of these two compounds indicated that the carbonyl of C-1 and the hydroxymethyl of C-18 in **7** were replaced by two methyls in **3**. This assumption was evidenced by the COSY relationships from H_3_-1 (δ_H_ 1.67) to H-2 (δ_H_ 5.29) and between H-13 (δ_H_ 2.48) and H_3_-18 (δ_H_ 1.13), in addition to the HMBC interactions from H_3_-1 to C-2 (δ_C_ 123.7) and C-3 (δ_C_ 134.9) and from H_3_-18 to C-12 (δ_C_ 74.1), C-13 (δ_C_ 47.5), and C-14 (δ_C_ 179.7). In the NOESY spectrum, the cross-peaks from H-4 (δ_H_ 5.59) to H_2_-6 (δ_H_ 2.03, 1.79) confirmed the 4*E* geometry. It is noteworthy that the overlapped signals of H_3_-1 (δ_H_ 1.67) and H_3_-15 (δ_H_ 1.69) were unable to be used to determine the Δ^2^ configuration based on the NOESY correlations. Finally, the 2*E* geometry was assigned by the comparison of the ^13^C NMR data of C-1 (δ_C_ 13.7) and C-15 (δ_C_ 17.0) of **3** with those of a structurally partial identity of pteroenone (δ_C_ 13.6, C-1 and 16.6, C-15) [27]. Thus, **3** was elucidated to be 1-deoxo-1,18-dedioxy-12-*epi*-1233B, and named fusarisolin C.

Compound **4** showed the same molecular formula as that of **3** by the HRESIMS spectrum and their NMR spectroscopic data were nearly identical, indicating a structurally related analogue. Comparison of the NMR data, as well as the analyses of 2D NMR spectra (HSQC, COSY, and HMBC), established the structure of **4** to be a 2*Z* isomer of **3**. This assumption was recognized by the shielded chemical shifts of H_3_-1 (Δδ_H_ −0.17), the deshielded shift of Me-15 (Δδ_C_ +7.2), in association with the NOESY relationships from H-2 (δ_H_ 5.30) to H_3_-15 (δ_H_ 1.70) and from H-4 (δ_H_ 5.53) to H_2_-6 (δ_H_ 2.09, 1.86). The 2*Z* and 4*E* configuration of the double bond at Δ^2^ and Δ^4^ of **4** were unambiguously determined by the clear NOESY data, which further supported the 2*E* and 4*E* configurational assignments of **3**. Accordingly, the structure of **4** was established as (2*Z*)-fusarisolin C, and named fusarisolin D.

Compound **5** exhibited the sodium adduct ion peak at *m*/*z* 249.1104 in the HRESIMS spectrum, consistent with the molecular formula of C_12_H_18_O_4_. The ^1^H NMR spectrum exhibited three methyls (δ_H_ 1.26, 1.63, and 2.12), one methoxy (δ_H_ 3.70), two olefinic protons (δ_H_ 5.32 and 5.74), one methylene (δ_H_ 2.85), and one methine (δ_H_ 3.39) (Table 1). The ^13^C NMR spectrum showed 12 carbons including four olefinic carbons for two double bonds, two carbonyl carbons, in addition to six sp^3^ carbons for three methyls, one methoxy, one methylene, and one methine (Table 2). The HMBC correlations originating from H-2 (δ_H_ 5.74), H_3_-9 (δ_H_ 2.12), H_3_-10 (δ_H_ 1.63), H_3_-11 (δ_H_ 1.26), and the OMe (δ_H_ 3.70) to the corresponding carbons of C-1−C-8 established the alkyl chained structure. The NOESY correlations from H-2 to H_2_-4 (δ_H_ 2.85) and from H_3_-10 to H-7 (δ_H_ 3.39) indicated 2*E* and 5*E* configurations. According to the similar optical rotation (OR) values of **5** ($[\alpha {]}_{D}^{24}$ +14.5), and the structurally related (*S*,*E*)-callosobruchusic acid ($[\alpha {]}_{D}^{25}$ +10.1) [28], the sole stereogenic center of C-7 was assigned as *S* configuration. Consequently, the structure of **5** was established as (2*E*,5*E*,7*S*)-8-methoxy-3,5,7-trimethyl-8-oxoocta-2,5-dienoic acid, and named fusarisolin E.

Compound **6** exhibited the molecular formula of C_19_H_32_O_6_ as established by the HRESIMS spectrum of the sodium adduct ion peak (*m*/*z* 379.2094). The NMR data were nearly identical to those of **7** except for the presence of additional methoxyl signals (δ_H_ 3.71, δ_C_ 52.0). The location of methoxyl group at C-14 (δ_C_ 175.1) was corroborated by the HMBC interaction from the methoxyl protons (δ_H_ 3.71) to the carbonyl carbon of C-14. The NOESY cross-peaks from H_2_-6 (δ_H_ 1.89, 2.14) to H-4 (δ_H_ 5.76) and from H-4 to H-2 (δ_H_ 5.65) revealed the 2*E* and 4*E* configurations of the conjugated double bonds. Therefore, the structure of **6** was elucidated as 14-*O*-methyl-12-*epi*-1233B. Although **6** is commercially available, no physicochemical data could be found. Accordingly, its ^1^H and ^13^C NMR data (Table 1 and Table 2) are reported here for the first time.

**Figure 4:**
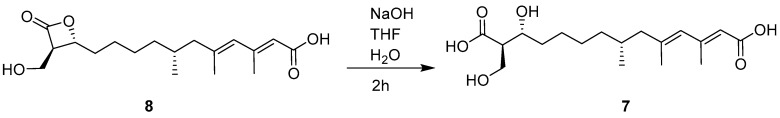
Alkaline hydrolysis of **8** to yield **7**.

In order to establish the absolute configuration of C-12 in **7**, compound **8** (1233A) was subjected to alkaline hydrolysis to provide **7** (Figure 4). According to the identical ^1^H- and ^13^C- NMR spectra and specific rotation data between hydrolysis product of **8** ($[\alpha {]}_{D}^{24}$ +7.2, MeOH) and **7** ($[\alpha {]}_{D}^{24}$ +6.7, MeOH) (Supplementary Materials Figure S8-1 and 8-2), **7** was concluded to have the opposite configuration at C-12 to that of 1233B, establishing **7** to be 12-*epi*-1233B [2]. Considering the absence of the ^13^C NMR spectroscopic data of 12-*epi*-1233B in the literature, the modern ^1^H- and ^13^C-NMR data of **7** are provided in Table S1 of the Supplementary Materials.

By comparing NMR spectroscopic data and specific rotations with those published in the literature, six known phenolics were determined to be dihydronaphthalenone B (**9**) [29], 2,3-dihydro-5-hydroxy-8-methoxy-2,4-dimethylnaphthol[1,2-b]furan-6,9-dione (**10**) [30], fusarubin (**11**) [31], methyl ether fusarubin (**12**) [32,33], solaniol (**13**) [34,35], and javanicin (**14**) [31].

**Figure 5:**
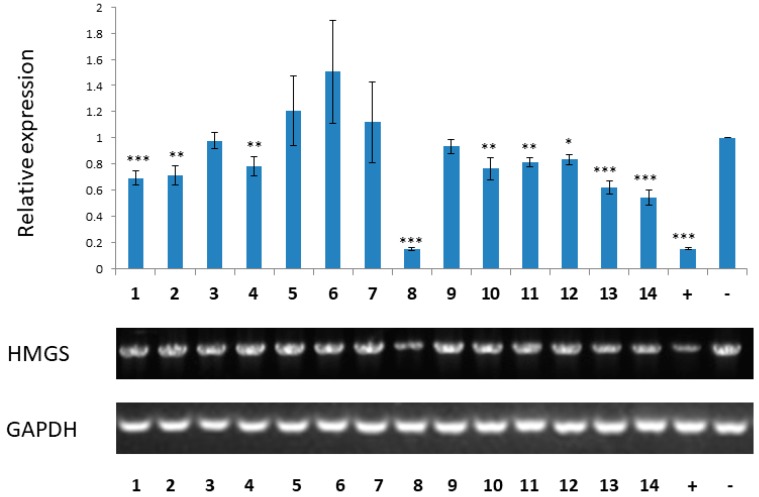
Inhibitory effects of **1** to **14** on the mRNA expression of HMG-CoA synthase. The gene expression level was determined by real-time polymerase chain reaction (RT-PCR). Dimethyl sulfoxide (DMSO) and abscisic acid were used as blank and positive controls, respectively. G lyceraldehyde-3-phosphate dehydrogenase (GAPDH) was used as reference gene. Values represent the mean ± SD of three independent experiments. * *p* < 0.05, ** *p* < 0.01, and *** *p* < 0.001.

Compounds **1** to **14** were evaluated for antifungal activities against tea pathogenic fungus *Pestalotiopsis theae.* Only compound **8** exhibited a potent effect with an ED_50_ value of 55 ± 4.0 μM, which was stronger than that of the positive control hexaconazole (ED_50_ = 68 ± 5.7 μM). Previously, 1233A (**8**) was reported to be a specific inhibitor of HMG-CoA synthase [3]. However, currently there is no commercially available *Pestalotiopsis theae* HMG-CoA synthase enzyme. It is known that synthase gene expression is positively correlated to synthase expression, i.e., the down/up regulation of gene expression leads to the lower/higher expression of protein. Therefore, all isolates were tested for the down-regulation of *Pestalotiopsis theae* HMG-CoA synthase gene expression by real-time polymerase chain reaction (RT-PCR) at the concentration of 10 µM, and abscisic acid and dimethyl sulfoxide (DMSO) were used as positive and blank controls [36]. As a result, compounds **1**, **8**, **13**, and **14** showed a significant effect, while **2**, **4**, **10**, and **11** exhibited a moderate effect, and **12** showed a weak effect (Figure 5). As in the case of **8**, compounds **1**, **13**, and **14** might also have potent inhibitory activity against HMG-CoA synthase, revealing their potential applications in regard to control of cholesterol biosynthesis.

## 3. Materials and Methods

### 3.1. General Experimental Procedures

Optical rotations were recorded on a Rudolph Autopol IV automatic polarimeter under 24 °C (Rudolph Research Analytical, Hackettstown, NJ, USA). The IR spectrum was recorded on a Bruker IFS-55 spectrometer (Bruker Optik BmbH, Ettlingen, Germany). The HRESIMS spectra were measured by a Waters Xevo G2 Q-TOF mass spectrometer (Waters, Milford, MA, USA). The NMR spectra were recorded on a Bruker Avance-400 FT MHz NMR spectrometer (Bruker, Fällanden, Switzerland). High performance liquid chromatography (HPLC) was carried out on an Alltech instrument equipped with UV detector (Series III, Alletch Inc., Nicholasville, Kentucky, USA). Thin-layer chromatography (TLC) analysis was performed on precoated silica gel plates (Jiangyou Silica Gel Development, Inc., Yantai, China). Column chromatography (CC) was performed on Sephadex LH-20, ODS, and silica gel, respectively.

### 3.2. Fungal Identification, Fermentation, and Extract

The fungus *Fusarium solani* H918 was isolated from mangrove sediments collected at the Zhangjiangkou Mangrove National Nature Reserve, Fujian, China. The internal transcribed spaces (ITS) region was amplified and sequenced by the general primers ITS1 and ITS4. The ITS region of the fungus was 576 bp DNA sequence (GenBank accession number: KY978584), which showed 99% identity to *Fusarium solani*. The strain was deposited at the China Center for Type Culture Collection (CCTCC) with the accession number of M 2017151.

Prior to the large-scale fermentation, the producing strain was incubated on a potato dextrose agar (PDA) plate medium under 25 °C for 3 days, and then the fresh mycelia were inoculated to 25 × 1 L Erlenmeyer flasks, each containing 80 g of rice and 120 mL of sea water. The fermentation was carried out under static conditions at 25 °C for 20 days. Following this, fermented cultures were extracted with EtOAc three times and concentrated under reduced pressure to get an organic extract. The extract was re-dissolved in MeOH and extracted with petroleum ether (PE) three times. The MeOH layer was evaporated under reduced pressure to get a defatted extract (6 g).

### 3.3. Isolation and Purification

The defatted extract was separated by an octadecylsilane (ODS) column using medium pressure liquid chromatography (MPLC) eluting with a gradient of MeOH-H_2_O (5:95→100:0) to get four fractions (Fr.1−Fr.4). Fraction Fr.2 (1.2 g) was chromatographed over silica gel CC using CHCl_3_-MeOH gradient elution (20:1→10:1) to get three subfractions (Fr.2-1−Fr.2-3). Subfraction Fr.2-1 was subjected to repeated silica gel CC eluting with PE-EtOAc (3:1) and PE-acetone (5:1) to yield **5** (2.2 mg) and **14** (2.7 mg). Subfraction Fr.2-2 was purified by CC over silica gel eluting with PE-EtOAc (3:1) to obtain **10** (11.2 mg) and **13** (9.8 mg). Subfraction Fr.2-3 was subjected to CC on silica gel eluting with CHCl_3_-MeOH-formic acid (20:1:0.1) to yield **7** (42.6 mg), **11** (1.7 mg), and **12** (5.5 mg). Fraction Fr.3 (625 mg) was fractionated by CC over Sephadex LH-20 (MeOH) to get four subfractions (Fr.3-1−Fr.3-4). Subfraction Fr.3-1 was purified by Prep. TLC using CHCl_3_-MeOH (10:1) elution to obtain **9** (8.0 mg). Subfraction Fr.3-2 was subjected to CC over silica gel (PE-acetone-formic acid, 1:1:0.01) to get **6** (25.0 mg). Compound **8** (18.2 mg) was separated from subfraction Fr.3-3 by CC over silica gel eluting with PE-acetone (3:1). Fraction Fr.4 (862 mg) was subjected to CC over Sephadex LH-20 (MeOH) to yield three subfractions (Fr.4-1−Fr.4-3). Compounds **1** (4.2 mg) and **2** (1.4 mg) were separated from subfraction Fr.4-2 by Prep. TLC (CHCl_3_-acetone, 3:1). Subfraction Fr.4-3 was purified by CC on silica gel (PE-acetone, 2:1), followed by semipreparative HPLC (MeOH-H_2_O, 3:1) to provide **3** (4.6 mg) and **4** (22.0 mg).

Fusarisolin A (**1**): Colorless oil; $[\alpha {]}_{D}^{24}$ +0.8 (*c* 0.19, MeOH); UV (MeOH) *λ*_max_ (log *ε*) 204 (3.80), 270 (4.11) nm; IR (KBr) *ν*_max_ 2926, 2856, 1818, 1688, 1615, 1457, 1379, 1249, 1162, 1022 cm^−1^; ^1^H and ^13^C NMR data, see Table 1 and Table 2; HRESIMS *m*/*z* 367.2446 [M + H]^+^ (calcd for C_21_H_35_O_5_, 367.2426).

Fusarisolin B (**2**): Colorless oil; $[\alpha {]}_{D}^{24}$ −5.3 (*c* 0.05, MeOH); UV (MeOH) *λ*_max_ (log *ε*) 206 (3.87), 268 (3.99) nm; ^1^H and ^13^C NMR data, see Table 1 and Table 2; HRESIMS *m*/*z* 365.2299 [M − H]^−^ (calcd for C_21_H_33_O_5_, 365.2328).

Fusarisolin C (**3**): Colorless oil; $[\alpha {]}_{D}^{24}$ −15.2 (*c* 0.04, MeOH); ^1^H and ^13^C NMR data, see Table 1 and Table 2; HRESIMS *m*/*z* 295.2256 [M − H]^−^ (calcd for C_18_H_31_O_3_, 295.2273).

Fusarisolin D (**4**): Colorless oil; $[\alpha {]}_{D}^{24}$ −19.8 (*c* 0.1, MeOH); ^1^H and ^13^C NMR data, see Table 1 and Table 2; HRESIMS *m*/*z* 295.2256 [M − H]^−^ (calcd for C_18_H_31_O_3_, 295.2273).

Fusarisolin E (**5**): Colorless oil; $[\alpha {]}_{D}^{24}$ +14.5 (*c* 0.03, MeOH); UV (MeOH) *λ*_max_ (log *ε*) 216 (4.19), 314 (3.14) nm; ^1^H and ^13^C NMR data, see Table 1 and Table 2; HRESIMS *m*/*z* 249.1104 [M + Na]^+^ calcd for (C_12_H_18_O_4_Na, 249.1103).

14-*O*-Methyl-12-*epi*-1233B (**6**): Colorless oil; $[\alpha {]}_{D}^{24}$ +0.7 (*c* 0.67, MeOH); ^1^H and ^13^C NMR data, see Table 1 and Table 2; HRESIMS *m*/*z* 379.2094 [M + Na]^+^ (calcd for C_19_H_32_O_6_Na, 379.2097).

### 3.4. Preparation of MPA Esters of 1

Compound **1** (1.5 mg) and (*R*)-MPA (3.0 mg) were dissolved in CHCl_3_ (600 µL). Following this, *N*,*N*′-dicyclohexylcarbodiimide (DCC, 2.5 mg) and 4-dimethylaminopyridine (DMAP, 2.8 mg) were added and the mixture was stirred for 24 h. The final reaction products were purified by CC over silica gel (PE-acetone, 3:1) to give the (*R*)-MPA ester (**1a**, 1.8 mg). Similarly, (*S*)-MPA ester (**1b**, 2.0 mg) was obtained from the reaction mixture of **1** (1.5 mg) and (*S*)-MPA (3.0 mg).

(*R*)-MPA ester of **1** (**1a**): ^1^H NMR (CDCl_3_, 400 MHz) δ_H_ 7.36−7.44 (5H, m, phenyl protons), 5.73 (1H, br s, H-4), 5.70 (1H, br s, H-2), 4.81 (1H, s, CH of MPA), 4.47 (1H, dd, *J* = 12.0, 4.2 Hz, H-21a), 4.39 (1H, dd, *J* = 12.0, 6.0 Hz, H-21b), 4.06 (1H, dd, *J* = 7.8, 4.2 Hz, H-14), 3.42 (3H, s, OMe of MPA), 2.25 (3H, s, Me-17), 2.10 (1H, dd, *J* = 13.7, 4.9 Hz, H-6a), 1.94 (1H, m, H-12a), 1.87 (1H, dd, *J* = 13.7, 6.3 Hz, H-6b), 1.82 (3H, s, Me-18), 1.72 (1H, m, H-9a), 1.69 (1H, m, H-13), 1.66 (1H, m, H-7), 1.61 (1H, m, H-10a), 1.36 (1H, m, H-11a), 1.34 (1H, m, H-9b), 1.29 (1H, m, H-8a), 1.23 (1H, m, H-11b), 1.18 (2H, m, H-10b and 12b), 1.10 (1H, m, H-8b), 0.96 (3H, d, *J* = 6.6 Hz, Me-20), 0.84 (3H, d, *J* = 6.6 Hz, Me-19).

(*S*)-MPA ester of **1** (**1b**): ^1^H NMR (CDCl_3_, 400 MHz) δ_H_ 7.36−7.46 (5H, m, phenyl protons), 5.74 (1H, br s, H-4), 5.70 (1H, br s, H-2), 4.81 (1H, s, CH of MPA), 4.43 (1H, dd, *J* = 12.2, 3.7 Hz, H-21a), 4.38 (1H, dd, *J* = 12.2, 4.8 Hz, H-21b), 3.68 (1H, dd, *J* = 7.9, 4.2 Hz, H-14), 3.43 (3H, s, OMe of MPA), 2.26 (3H, s, Me-17), 2.09 (1H, dd, *J* = 13.2, 5.9 Hz, H-6a), 1.94 (1H, m, H-12a), 1.85 (1H, dd, *J* = 13.2, 4.6 Hz, H-6b), 1.82 (3H, s, Me-18), 1.72 (1H, m, H-9a), 1.65 (1H, m, H-7), 1.63 (1H, m, H-13), 1.61 (1H, m, H-10a), 1.35 (1H, m, H-9b), 1.30 (1H, m, H-11a), 1.27 (1H, m, H-8a), 1.21 (1H, m, H-11b), 1.18 (2H, m, H-10b and 12b), 1.08 (1H, m, H-8b), 0.89 (3H, d, *J* = 6.5 Hz, Me-20), 0.84 (3H, d, *J* = 6.6 Hz, Me-19).

### 3.5. Alkaline Hydrolysis of 8

A tetrahydrofuran (THF) solution (100 μL) of **8** (5.0 mg, 0.015 mmol) was mixed with NaOH (2.5 mg, 0.0625 mmol) and H_2_O (400 μL). This reaction mixture was left at room temperature for 2 h, and then the pH value was adjusted to 3–4 with hydrochloric acid (1 M). After being extracted with CHCl_3_ three times, the extract was subjected to CC over ODS eluting with MeOH/H_2_O (7:3) to yield **7** (3.0 mg).

### 3.6. Antifungal Assay

Antifungal assay against tea pathogenic fungus *Pestalotiopsis theae* HQ832793, isolated from foliar lesions of tea leaf, was performed in PDA Petri plates according to a previously described method [37]. In brief, a 0.6 cm diameter piece of tested fungal strains cylinder agar was placed on the center, and sterile blank paper discs (0.5 cm diameter) were placed at a distance of 2 cm away from the growing mycelial colony. The tested compounds (100 μg/mL, DMSO solution) were added to each paper disc. DMSO and hexaconazole were used as blank and positive controls, respectively. These plates were incubated at 28 °C until mycelial growth enveloped the discs including the control disc. The experiment was repeated three times.

### 3.7. ED_50_ Detection

As reported previously [38], different concentrations of the DMSO dissolved 1233A and hexaconazole were mixed with a PDA medium and poured into a set of PDA Petri plates. The *Pestalotiopsis theae* mycelial disk (5 mm) was placed in the center of each treated Petri dish and incubated at 28 °C. All treatments were quadruplicated against each fungus. DMSO and hexaconazole were used as blank and positive controls, respectively. The ED_50_ value was calculated statistically by Probit analysis.

### 3.8. Total RNA Isolation

*Pestalotiopsis theae* cells were cultured in a PDA medium for 3 days at 28 °C, then treated with tested compounds for 16 h. Cells were harvested by centrifugation at 6000 rpm for 5 min, and then homogenized in liquid nitrogen. Total RNA was extracted with Spin Column Fungal Total RNA Purification Kit (Sangon Biotech, Shanghai, China).

### 3.9. RT-PCR Analysis of HMG-CoA Synthase Gene Expression

The inhibitory effects of the tested compounds at 10 µM (DMSO dissolution) on the mRNA expression of HMG-CoA synthase in *Pestalotiopsis theae* cells were analyzed by RT-PCR. DMSO and abscisic acid (10 µM) were used as blank and positive controls, respectively. The expression of mRNA transcripts of HMG-CoA synthase (forward: TACTCG CTCACCTGCTACAC; reverse: GCGTACGACTTCTGGACGAC) and GAPDH (forward: CATGTCCATGCGTGTCCCTA; reverse: CAGTGGAGACAACCTCGTCC) was determined by RT-PCR. The cDNA was synthesized from total RNA using PrimeScript RT reagent kit with gDNA Eraser (Takara, Japan). TaKaRa SYBR^®^ Premix Ex Taq™ II (Takara, Japan) and Stepone Real-Time PCR Detection System (Applied Biosystems, Foster City, CA, USA) were used for RT-PCR analysis. The values are expressed as the mean ± SD for three triplicate experiments.

## 4. Conclusions

The present work reported five new (**1** to **5**) and nine known (**6** to **14**) compounds from the marine-derived fungus *Fusarium solani* H918. Fusarisolins A (**1**) and B (**2**), two novel 21-carbon polyketides featuring a rare β- and γ-lactone unit, respectively, were found for the first time in nature. Compounds **1**, **8**, **13**, and **14** showed significant down-regulation HMG-CoA synthase gene expression. In addition, compound **8** exhibited potent inhibitory activity against tea pathogenic fungus *Pestalotiopsis theae*, revealing that it might be a potential lead compound for the development of an antifungal agrochemical after structural modification.

## Figures and Tables

**Table 1:** ^1^H NMR data for compounds **1** to **6** at 400 MHz (δ in ppm, *J* in parenthesis with Hz).

No.	1 ^a^	2 ^a^	3 ^a^	4 ^a^	5 ^b^	6 ^a^
1			1.67, d (7.0)	1.50, dm (6.8)		
2	5.65, br s	5.65, br s	5.29, q (7.0)	5.30, qq (6.8, 1.2)	5.74, br s	5.65, br s
4	5.76, br s	5.76, br s	5.59, br s	5.53, br s	2.85, s	5.76, br s
6	2.13, dd (13.2, 6.2)	2.14, dd (13.2, 7.2)	2.03, dd (12.8, 6.4)	2.09, dd (13.2, 6.3)	5.32, d (8.7)	2.14, dd (13.1, 6.4)
	1.88, dd (13.2, 8.1)	1.89, dd (13.2, 8.1)	1.79, m	1.86, ddd (13.2, 8.3, 0.8)		1.89, dd (13.1, 8.2)
7	1.70, m	1.71, m	1.65, m	1.66, m	3.39, m	1.71, m
8	1.35, m; 1.15, m	1.34, m; 1.15, m	1.36, m; 1.12, m	1.38, m; 1.15, m		1.36, m; 1.16, m
9	1.44, m	1.34, m	1.36, m	1.38, m	2.12, s	1.37, m
10	1.33, m	1.34, m	1.49, m; 1.37, m	1.50, m; 1.38, m	1.63, s	1.48, m; 1.37, m
11	1.33, m	1.42, m	1.52, m; 1.40, m	1.53, m; 1.40, m	1.26, d (6.8)	1.53, m; 1.45, m
12	1.44, m; 1.18, m	1.72, m	3.71, t (6.9)	3.70, t (6.9)		3.79, m
13	1.82, m		2.48, m	2.48, m		2.67, ddd (8.5, 6.3, 5.4)
14	4.31, dd (8.4, 4.2)	2.16, br d (10.3)				
15	3.53, q (4.2)	3.02, m	1.69, s	1.70, m		2.21, d (1.2)
16			1.71, s	1.54, d (1.3)		1.82, d (1.2)
17	2.20, s	2.21, s	0.84, d (6.6)	0.88, d (6.6)		0.87, d (6.6)
18	1.82, s	1.82, s	1.13, d (6.9)	1.13, d (7.0)		3.82, dd (10.9, 8.7)
						3.73, dd (10.9, 5.3)
19	0.87, d (6.6)	0.87, d (6.5)				
20	1.04, d (6.5)	1.38, s				
21	3.91, dd (11.9, 4.5)	3.87, dd (11.0, 4.6)				
	3.77, dd (11.9, 3.8)	3.72, dd (11.0, 3.6)				
OMe					3.70, s	3.71, s

^a^ Measured in CD_3_OD; ^b^ Measured in CDCl_3_.

**Table 2:** ^13^C NMR spectroscopic data for **1** to **6** at 100 MHz (δ in ppm).

No.	1 ^a^	2 ^a^	3 ^a^	4 ^a^	5 ^b^	6 ^a^
1	171.0, C	172.1, C	13.7, CH_3_	15.3, CH_3_	171.7, C	170.6, C
2	119.1, CH	119.1, CH	123.7, CH	121.8, CH	116.6, CH	118.8, CH
3	155.2, C	155.0, C	134.9, C	135.3, C	160.1, C	155.5, C
4	130.6, CH	130.6, CH	131.6, CH	126.8, CH	51.1, CH_2_	130.6, CH
5	142.2, C	142.1, C	135.1, C	137.2, C	133.5, C	142.3, C
6	49.9, CH_2_	49.9, CH_2_	49.8, CH_2_	48.8, CH_2_	128.0, CH	50.0, CH_2_
7	32.1, CH	32.2, CH	32.1, CH	32.1, CH	38.9, CH	32.1, CH
8	37.8, CH_2_	37.9, CH_2_	38.0, CH_2_	38.0, CH_2_	175.7, C	37.9, CH_2_
9	27.9, CH_2_	28.9, CH_2_	28.2, CH_2_	28.1, CH_2_	17.9, CH_3_	32.1, CH_2_
10	27.8, CH_2_	31.2, CH_2_	27.0, CH_2_	27.0, CH_2_	15.9, CH_3_	26.9, CH_2_
11	31.0, CH_2_	24.8, CH_2_	35.0, CH_2_	35.0, CH_2_	18.4, CH_3_	36.1, CH_2_
12	32.4, CH_2_	42.6, CH_2_	74.1, CH	74.1, CH		71.0, CH
13	38.1, CH	86.5, C	47.5, CH	47.5, CH		56.2, CH
14	80.2, CH	36.5, CH_2_	179.7, C	179.5, C		175.1, C
15	58.2, CH	44.6, CH	17.0, CH_3_	24.2, CH_3_		19.9, CH_3_
16	172.1, C	179.5, C	17.9, CH_3_	17.8, CH_3_		18.5, CH_3_
17	19.8, CH_3_	19.8, CH_3_	20.0, CH_3_	19.9, CH_3_		19.8, CH_3_
18	18.5, CH_3_	18.5, CH_3_	13.9, CH_3_	13.8, CH_3_		61.6, CH_2_
19	19.9, CH_3_	19.9, CH_3_				
20	15.3, CH_3_	25.5, CH_3_				
21	58.3, CH_2_	61.0, CH_2_				
OMe					51.9, CH_3_	52.0, CH_3_

^a^ Measured in CD_3_OD; ^b^ Measured in CDCl_3_.

## Supplementary Materials

The following are available online at http://www.mdpi.com/1660-3397/17/2/125/s1, Figures S1-1–S8-2 and Table S1: The 1D and 2D NMR spectra of **1**–**7**, ^1^H and ^13^C NMR data for **7**.
