# Supplementary material for: Fusarisolins A–E, Polyketides from the Marine-Derived Fungus Fusarium solani H918
Source: Mar Drugs. 2019 Feb 20;17(2):125. doi: 10.3390/md17020125 (PMC6410219; doi:10.3390/md17020125)
Supplement: Supplementary file 1 [file marinedrugs-17-00125-s001.pdf]

Supporting Information

Fusarisolins A−E, Polyketides from the Marine-Derived Fungus *Fusarium solani* H918

Siwen Niu, Xi-Xiang Tang, Zuowang Fan, Jin-Mei Xia, Chun-Lan Xie, and Xian-Wen Yang *

State Key Laboratory Breeding Base of Marine Genetic Resources, Key Laboratory of Marine Genetic Resources, Fujian Key Laboratory of Marine Genetic Resources, South China Sea Bio-Resource Exploitation and Utilization Collaborative Innovation Center, Third Institute of Oceanography, Ministry of Natural Resources, 184 Daxue Road, Xiamen 361005, People's Republic of China

Contents

Figure S1-1 ^{1}H NMR Spectrum of **1** in CD_3OD (400 MHz)

Figure S1-2 ^{13}C Spectrum of **1** in CD_3OD (100 MHz)

Figure S1-3 HSQC Spectrum of **1** in CD_3OD

Figure S1-4 COSY Spectrum of **1** in CD_3OD

Figure S1-5 HMBC Spectrum of **1** in CD_3OD

Figure S1-6 NOESY Spectrum of **1** in CD_3OD

Figure S1-7 ^{1}H NMR Spectrum of *R*-MPA ester of **1** (**1a**) in $CDCl_3$ (400 MHz)

Figure S1-8 ^{1}H NMR Spectrum of *S*-MPA ester of **1** (**1b**) in $CDCl_3$ (400 MHz)

Figure S1-9 IR Spectrum of **1**

Figure S2-1 ^{1}H NMR Spectrum of **2** in CD_3OD (400 MHz)

Figure S2-2 ^{13}C Spectrum of **2** in CD_3OD (100 MHz)

Figure S2-3 HSQC Spectrum of **2** in CD_3OD

Figure S2-4 COSY Spectrum of **2** in CD_3OD

Figure S2-5 HMBC Spectrum of **2** in CD_3OD

Figure S2-6 NOESY Spectrum of **2** in CD_3OD

Figure S3-1 ^{1}H NMR Spectrum of **3** in CD_3OD (400 MHz)

Figure S3-2 ^{13}C Spectrum of **3** in CD_3OD (100 MHz)

Figure S3-3 HSQC Spectrum of **3** in CD_3OD

Figure S3-4 COSY Spectrum of **3** in CD_3OD

Figure S3-5 HMBC Spectrum of **3** in CD_3OD

Figure S3-6 NOESY Spectrum of **3** in CD_3OD

Figure S4-1 ^{1}H NMR Spectrum of **4** in CD_3OD (400 MHz)

Figure S4-2 ^{13}C Spectrum of **4** in CD_3OD (100 MHz)

Figure S4-3 HSQC Spectrum of **4** in CD_3OD

Figure S4-4 COSY Spectrum of **4** in CD_3OD

Figure S4-5 HMBC Spectrum of **4** in CD_3OD

Figure S4-6 NOESY Spectrum of **4** in CD_3OD

Figure S5-1 ^{1}H NMR Spectrum of **5** in $CDCl_3$ (400 MHz)

Figure S5-2 ^{13}C Spectrum of **5** in $CDCl_3$ (100 MHz)

Figure S5-3 HSQC Spectrum of **5** in $CDCl_3$

Figure S5-4 COSY Spectrum of **5** in $CDCl_3$

Figure S5-5 HMBC Spectrum of **5** in $CDCl_3$

Figure S5-6 NOESY Spectrum of **5** in $CDCl_3$

Figure S6-1 ^{1}H NMR Spectrum of **6** in CD_3OD (400 MHz)

Figure S6-2 ^{13}C Spectrum of **6** in CD_3OD (100 MHz)

Figure S7-1 ^{1}H NMR Spectrum of **7** in CD_3OD (400 MHz)

Figure S7-2 ^{13}C Spectrum of **7** in CD_3OD (100 MHz)

Figure S8-1 Comparison of ^{1}H NMR Spectra of **7** and hydrolysis product of **8** (CD_3OD, 400 MHz)

Figure S8-2 Comparison of ^{13}C NMR Spectra of **7** and hydrolysis product of **8** (CD_3OD, 100 MHz)

Table S1. ^{1}H (400 MHz) and ^{13}C (100 MHz) NMR Data for **7** in CD_3OD

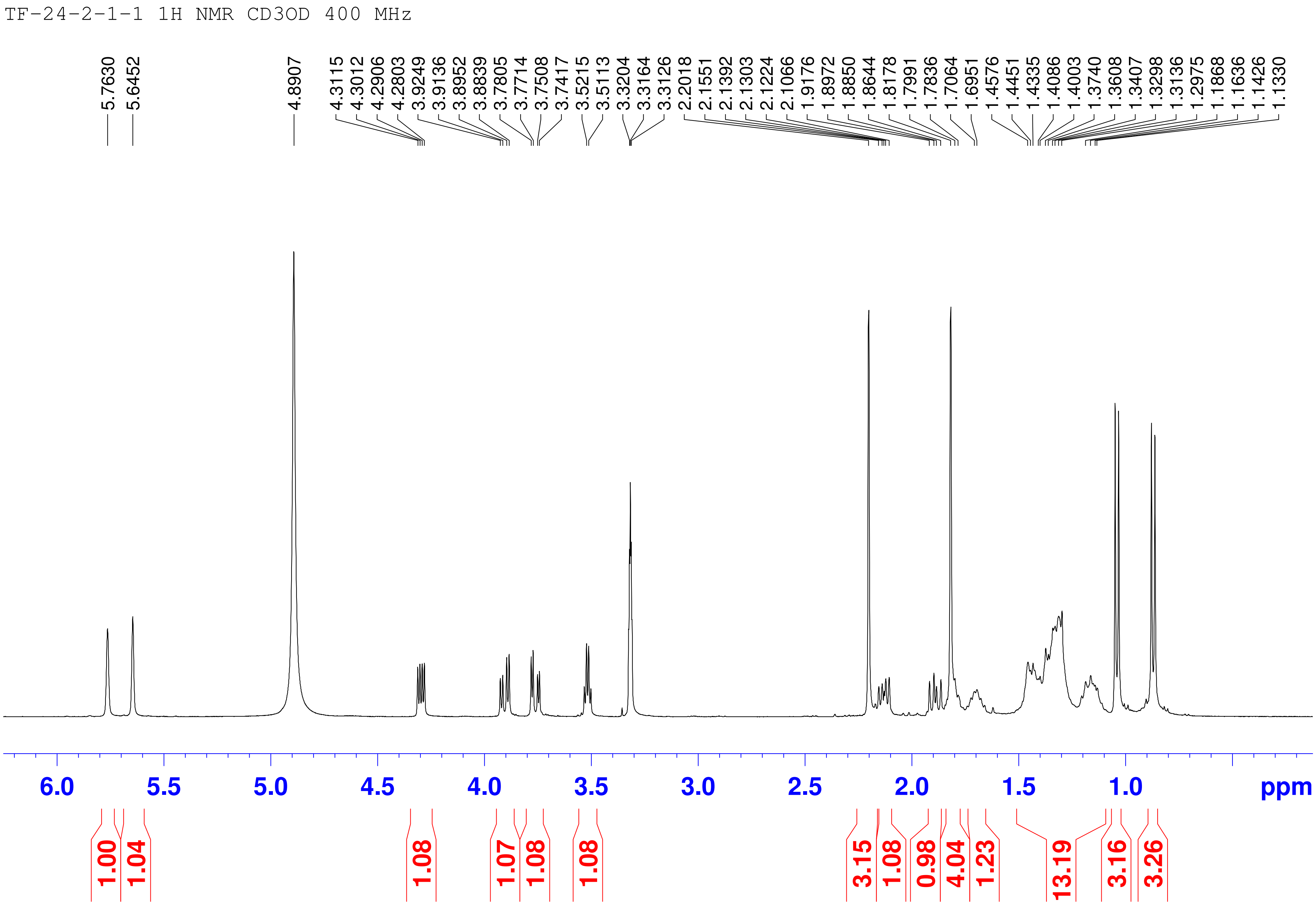

Figure S1-1 ^{1}H NMR Spectrum of **1** in CD_3OD (400 MHz)

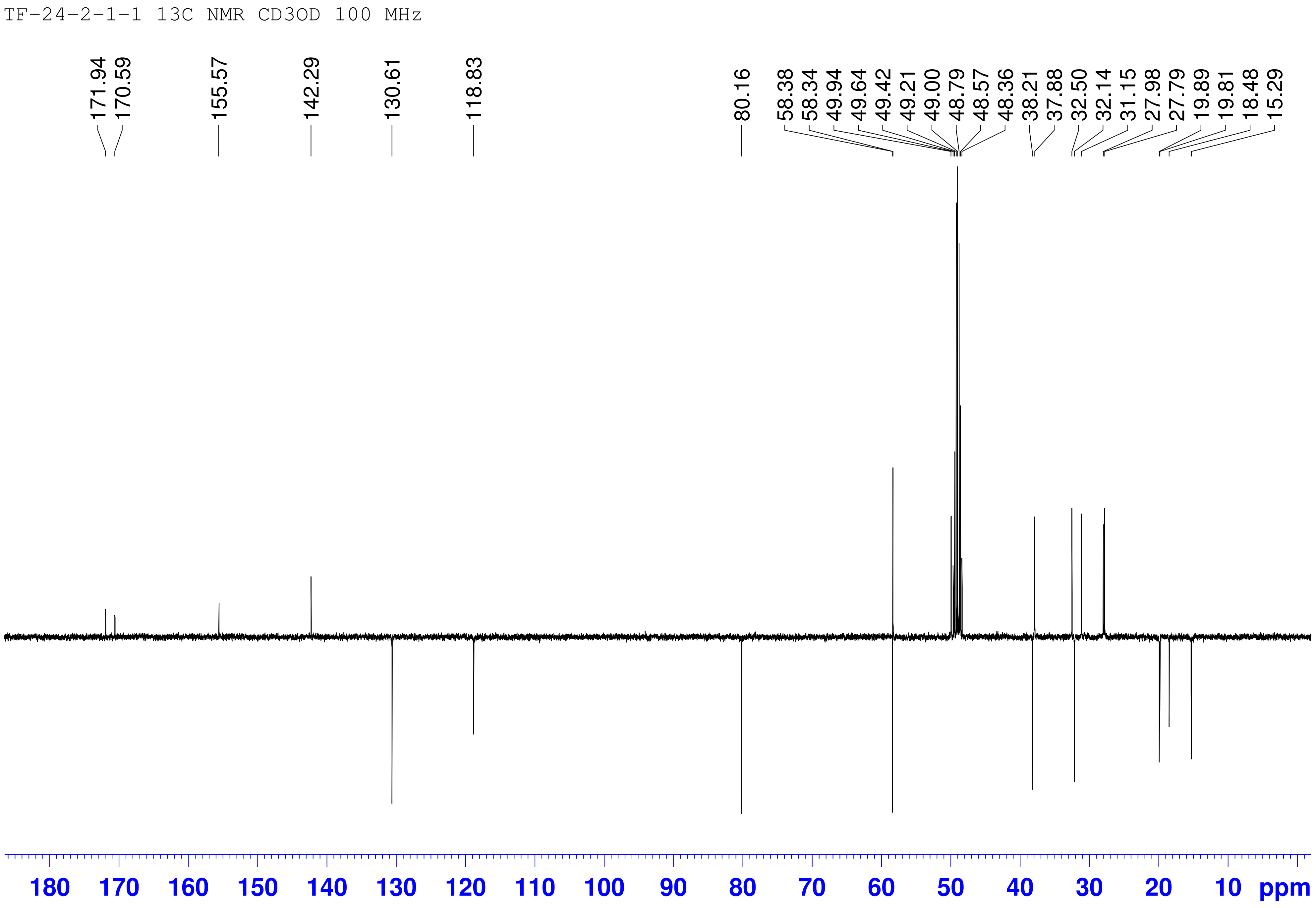

Figure S1-2 ^{13}C Spectrum of **1** in CD_3OD (100 MHz)

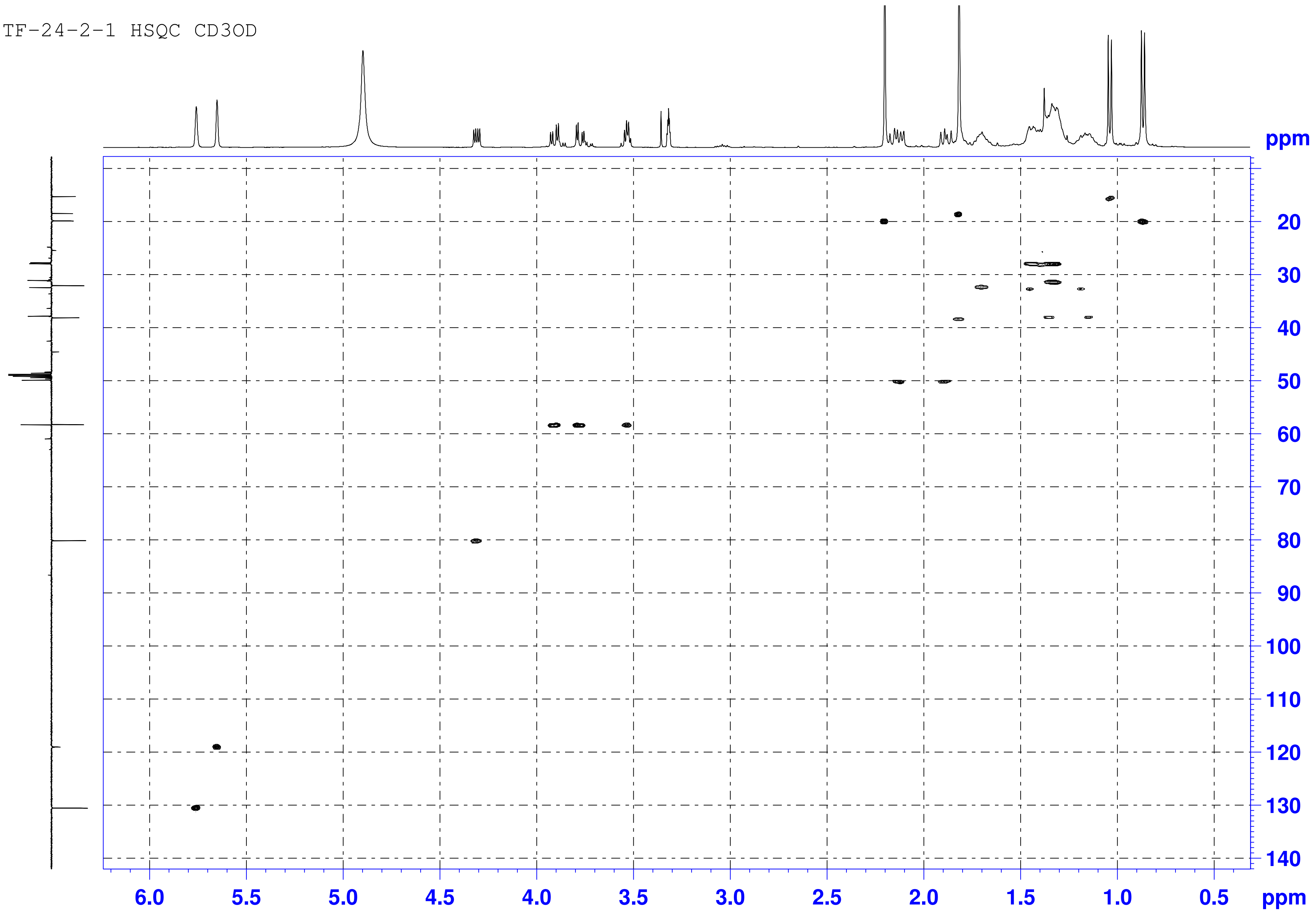

Figure S1-3 HSQC Spectrum of **1** in CD_3OD

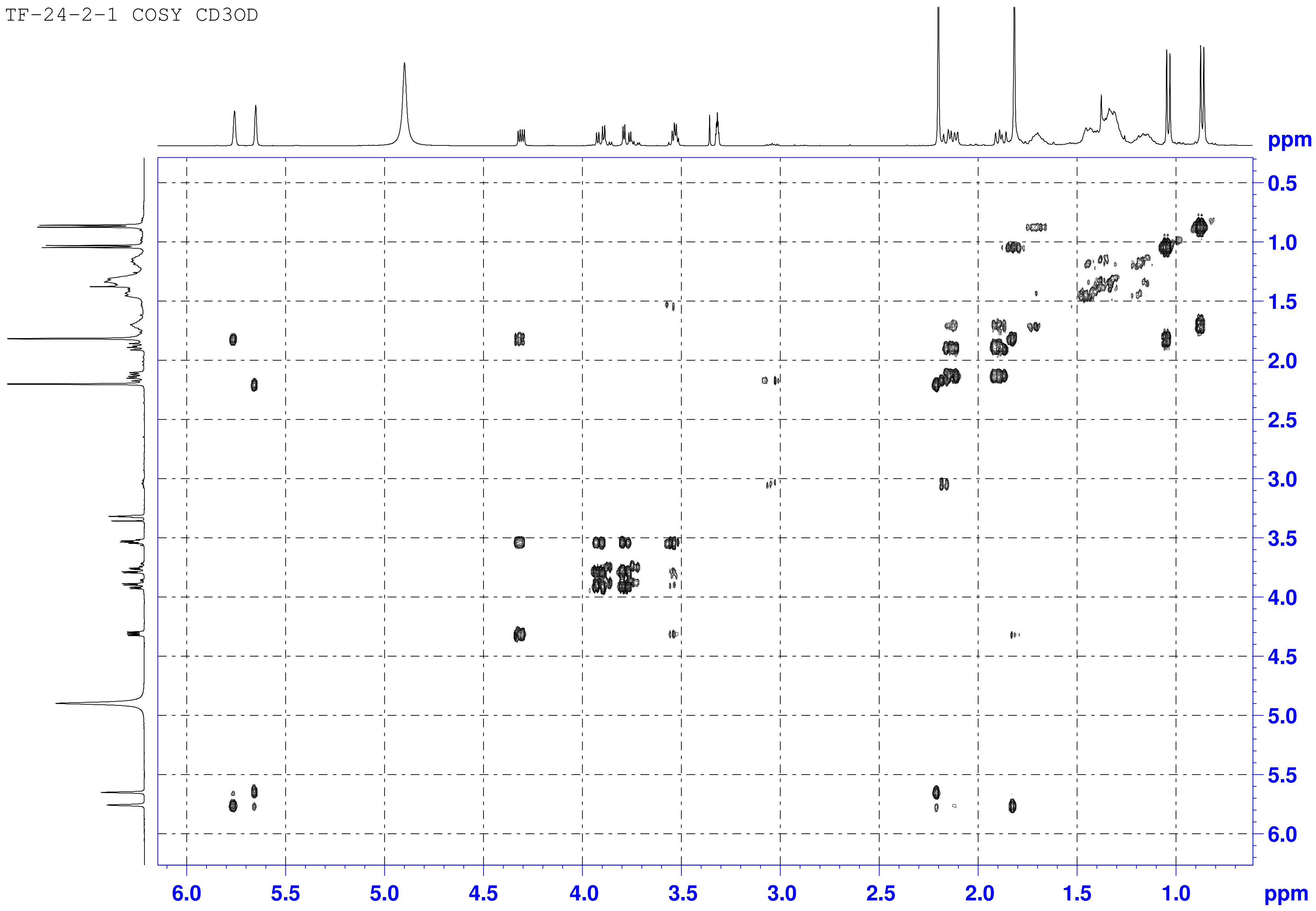

Figure S1-4 COSY Spectrum of **1** in CD_3OD

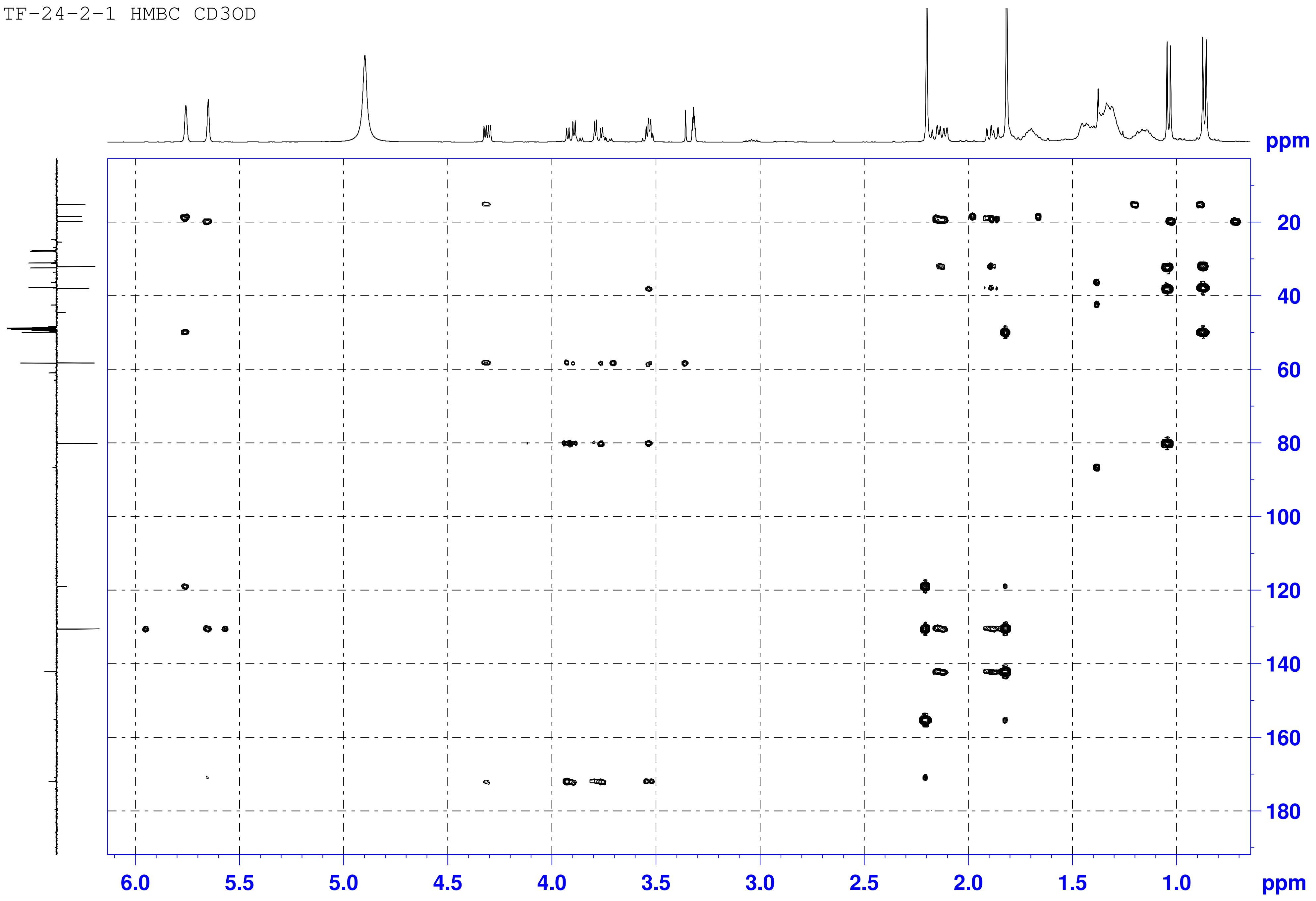

Figure S1-5 HMBC Spectrum of **1** in CD_3OD

TF-24-2-1

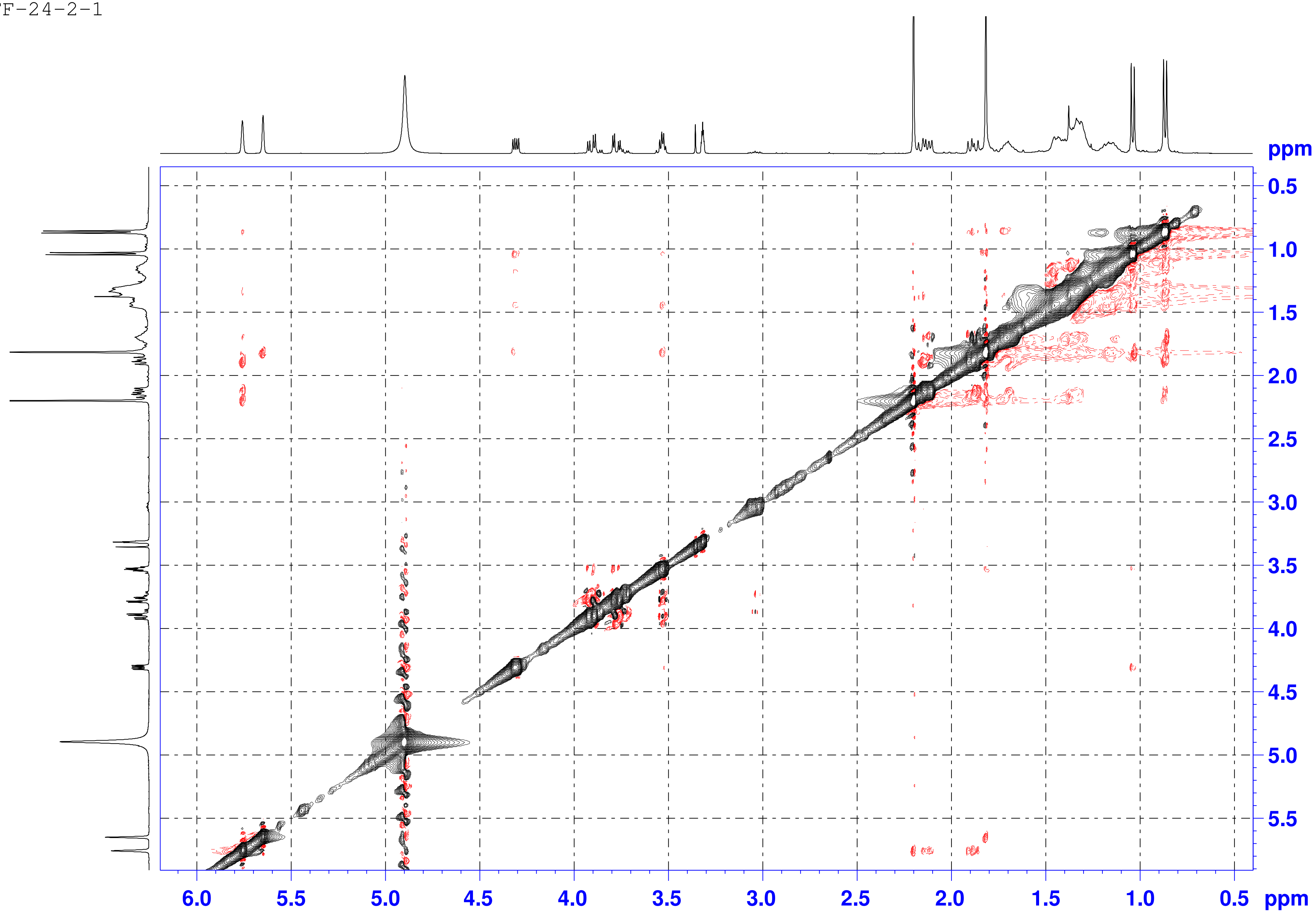

Figure S1-6 NOESY Spectrum of **1** in CD_3OD

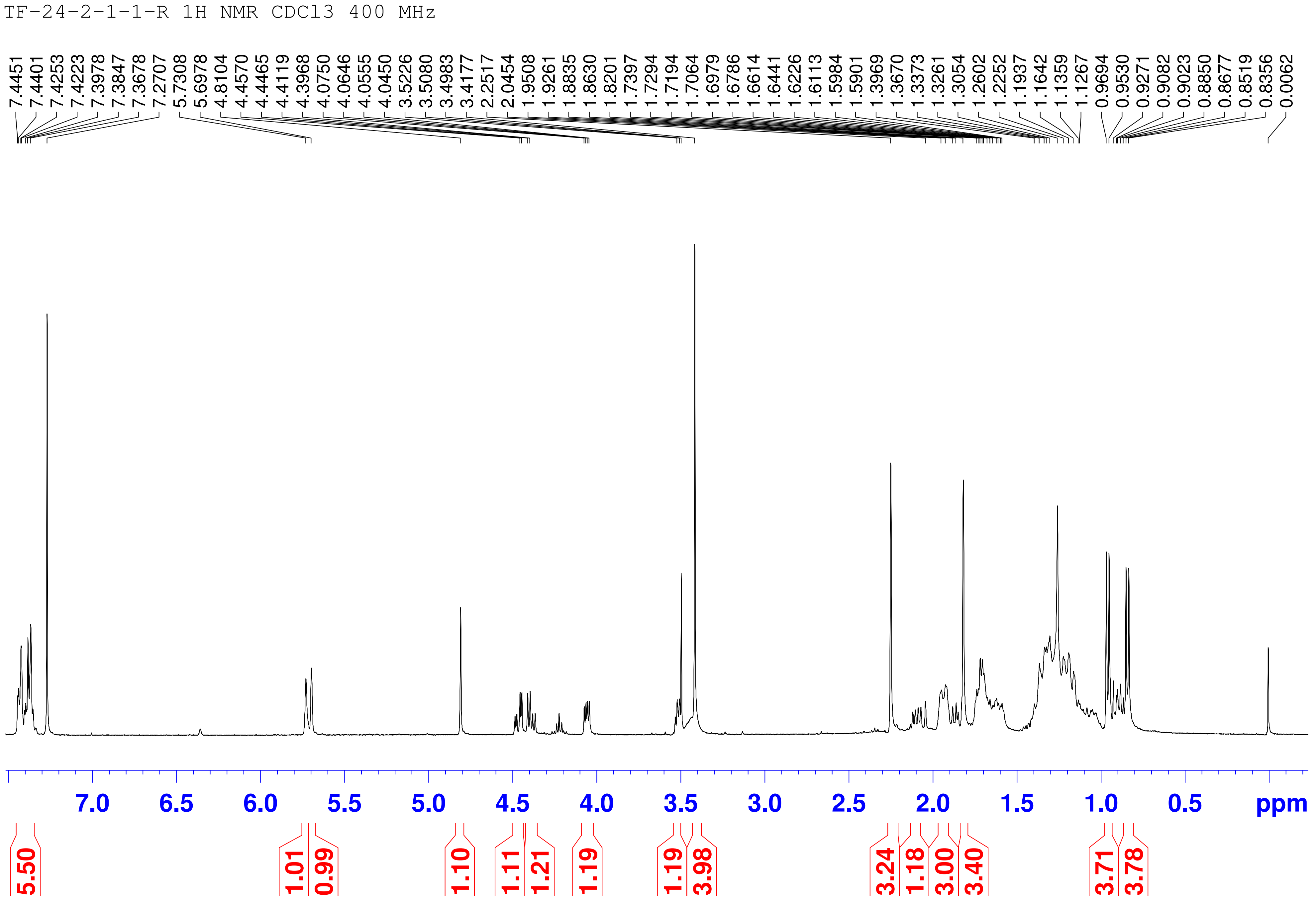

Figure S1-7 ^{1}H NMR Spectrum of R-MPA Ester of **1** (**1a**) in $CDCl_3$ (400 MHz)

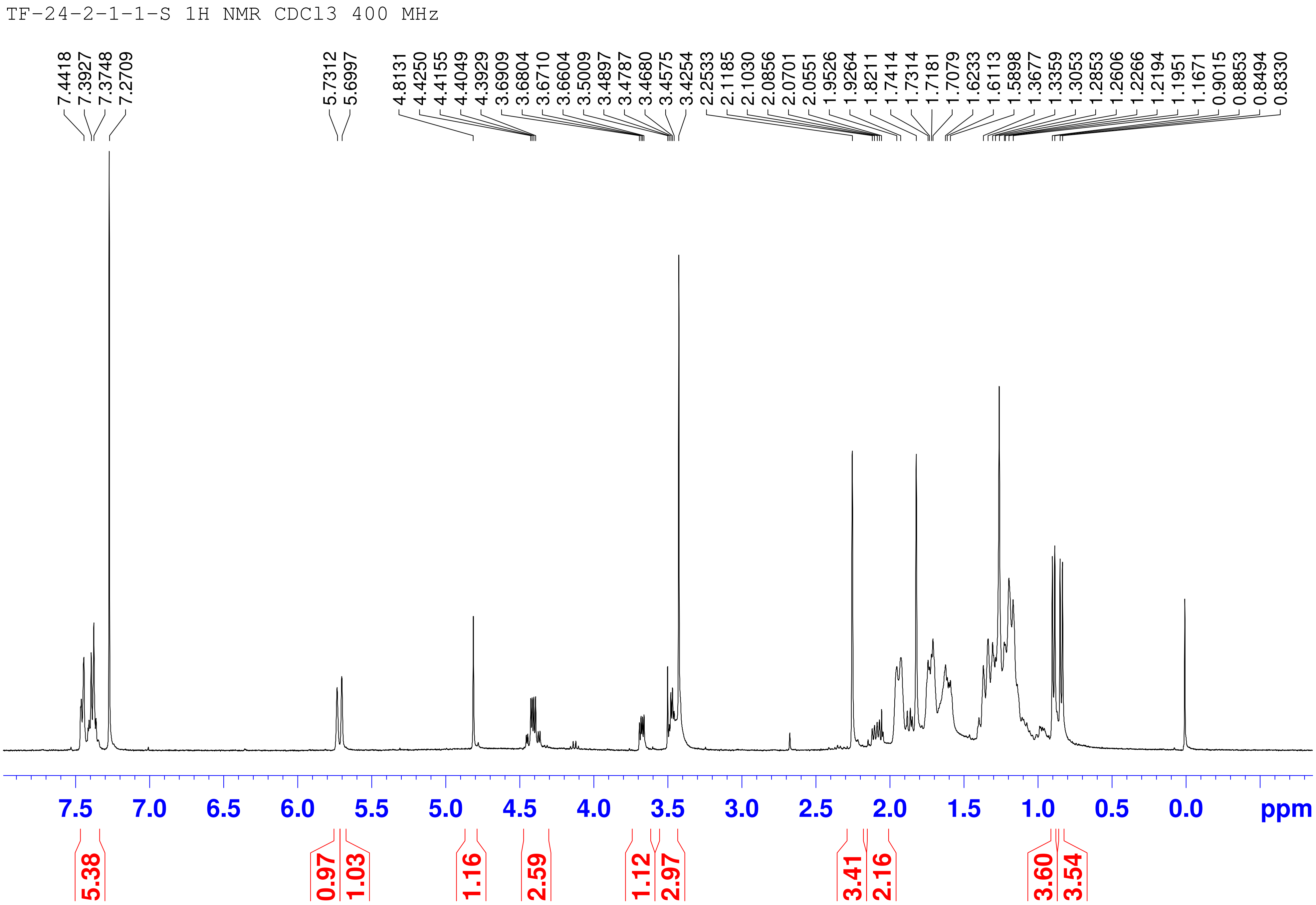

Figure S1-8 ^{1}H NMR Spectrum of S-MPA Ester of **1** (**1b**) in $CDCl_3$ (400 MHz)

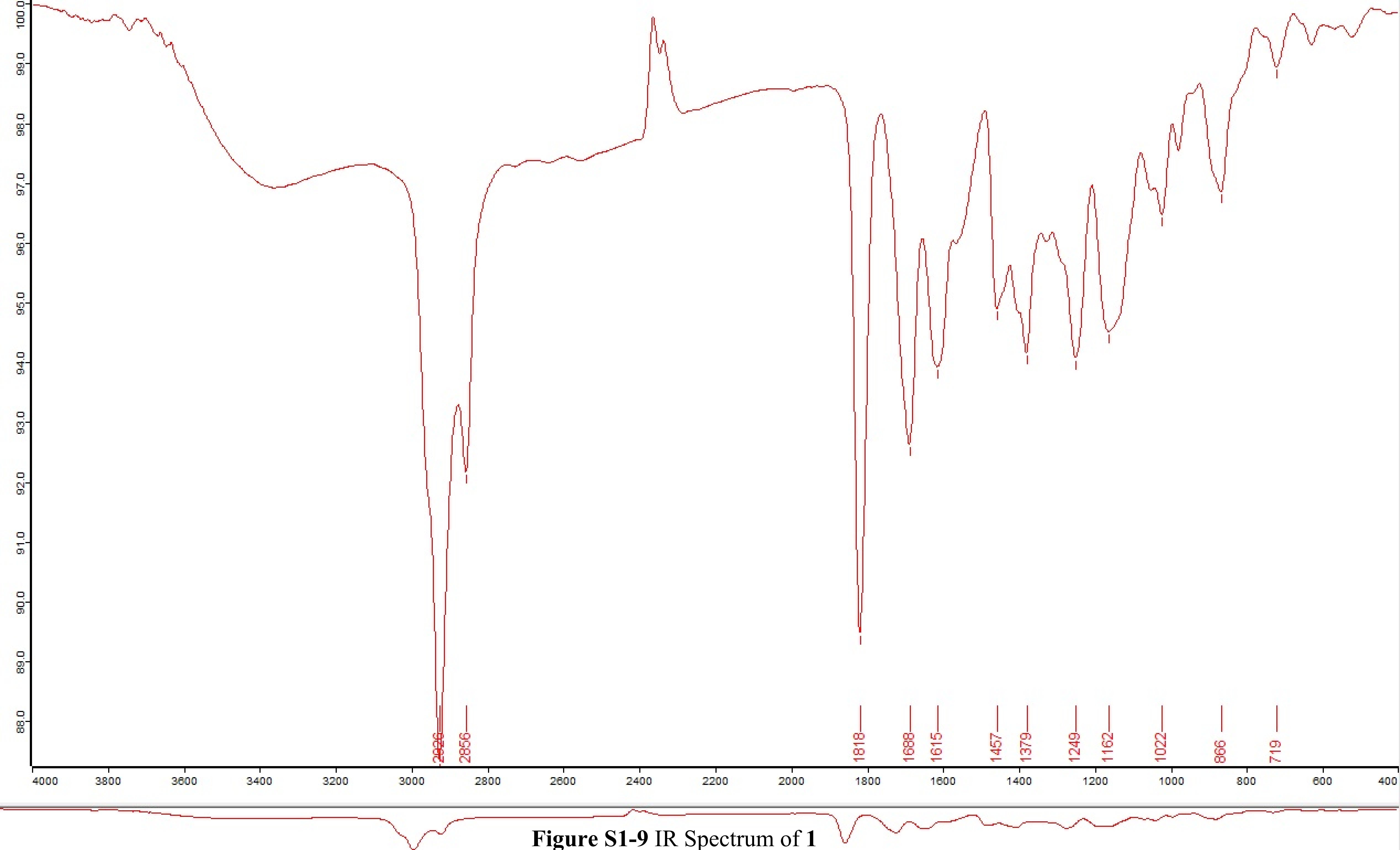

Figure S1-9 IR Spectrum of **1**

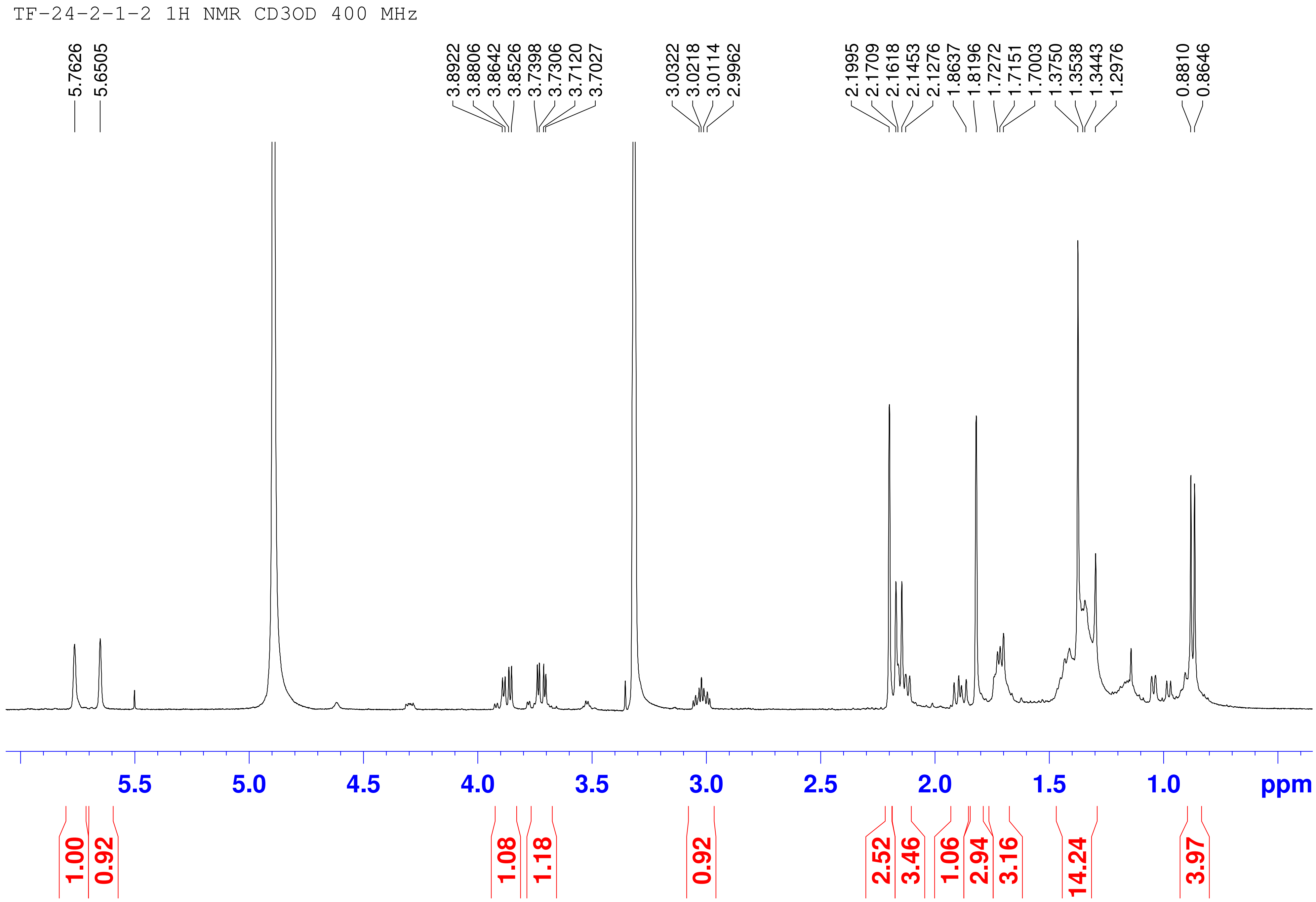

Figure S2-1 ^{1}H NMR Spectrum of **2** in CD_3OD (400 MHz)

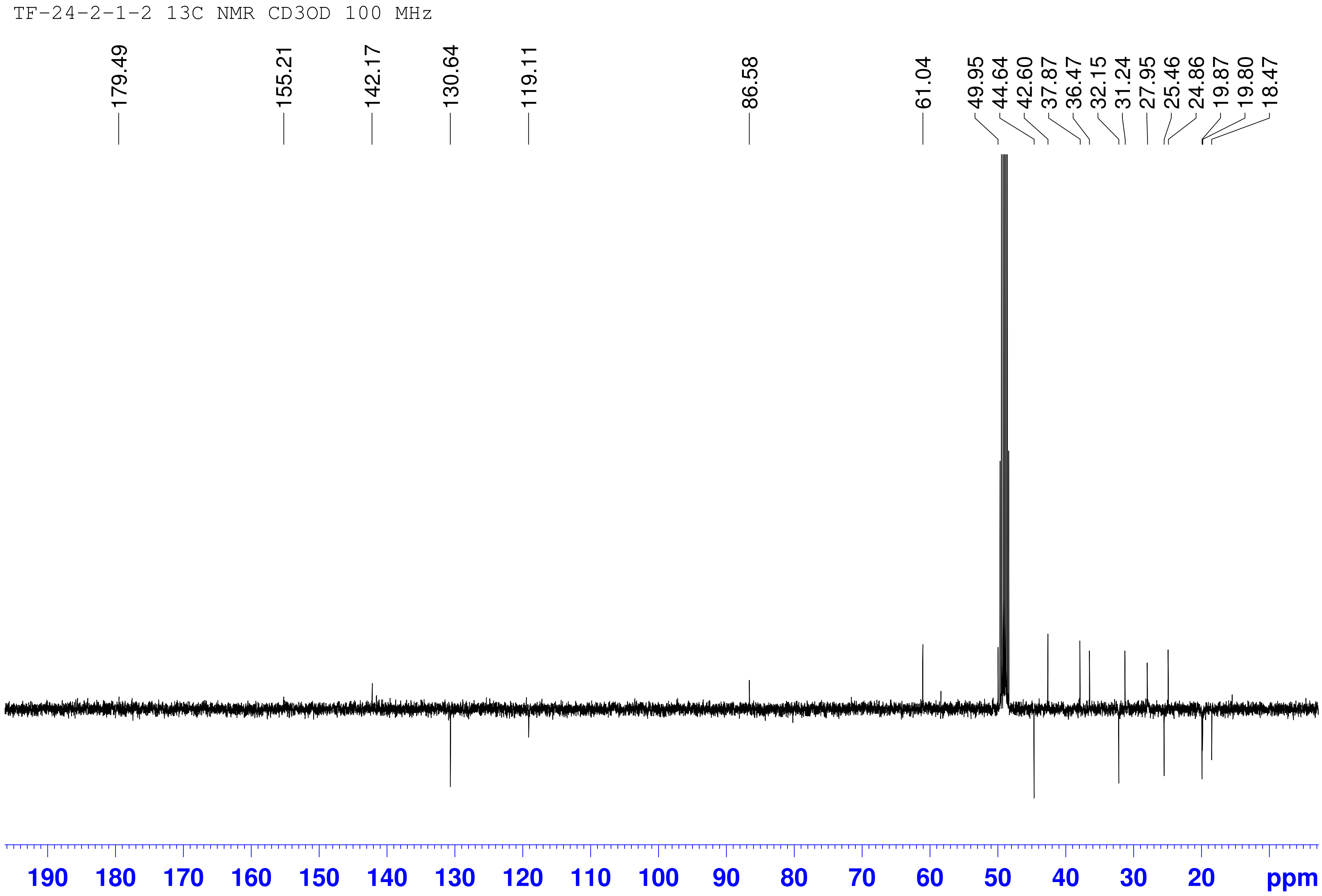

Figure S2-2 ^{13}C Spectrum of **2** in CD_3OD (100 MHz)

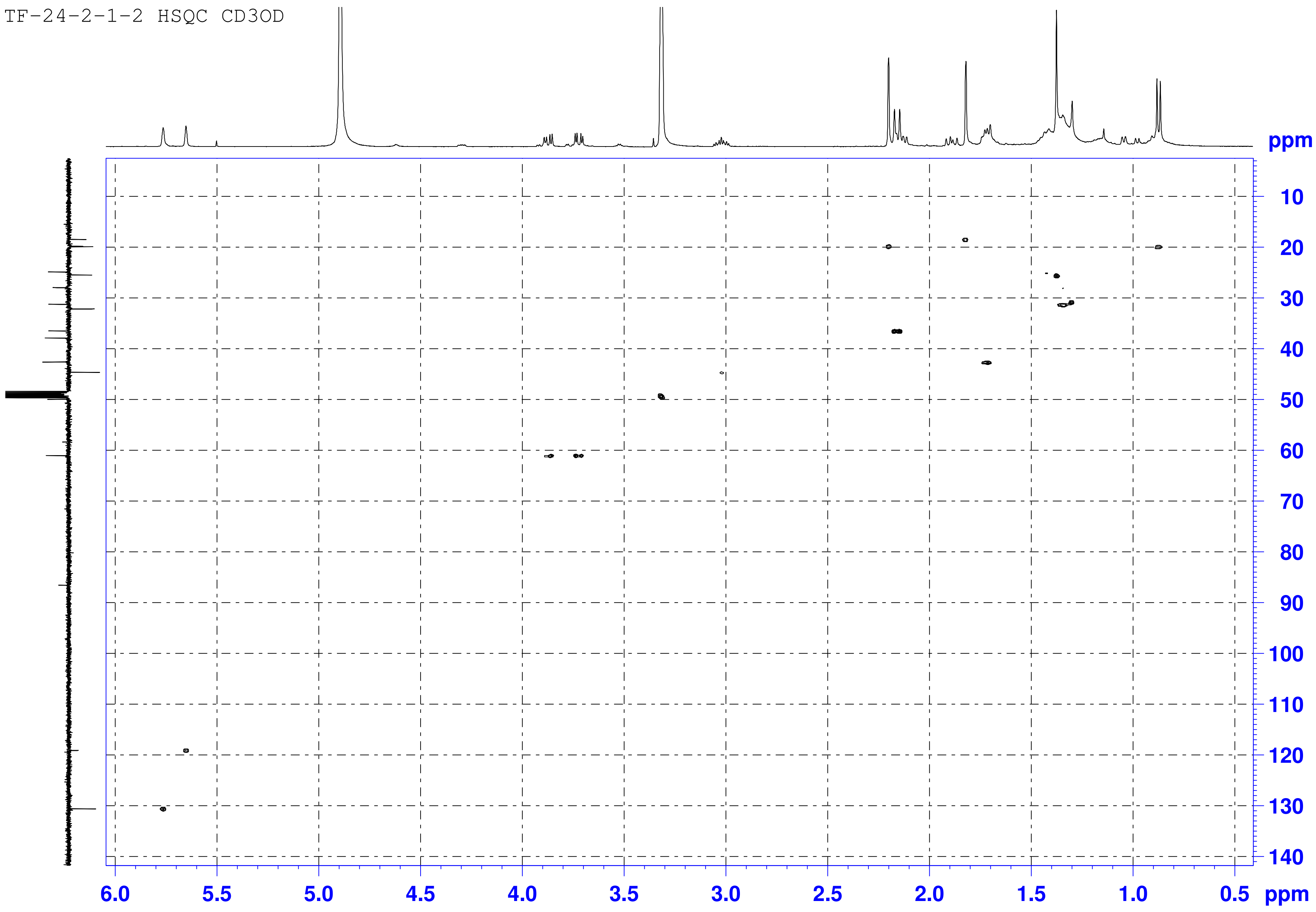

Figure S2-3 HSQC Spectrum of **2** in CD_3OD

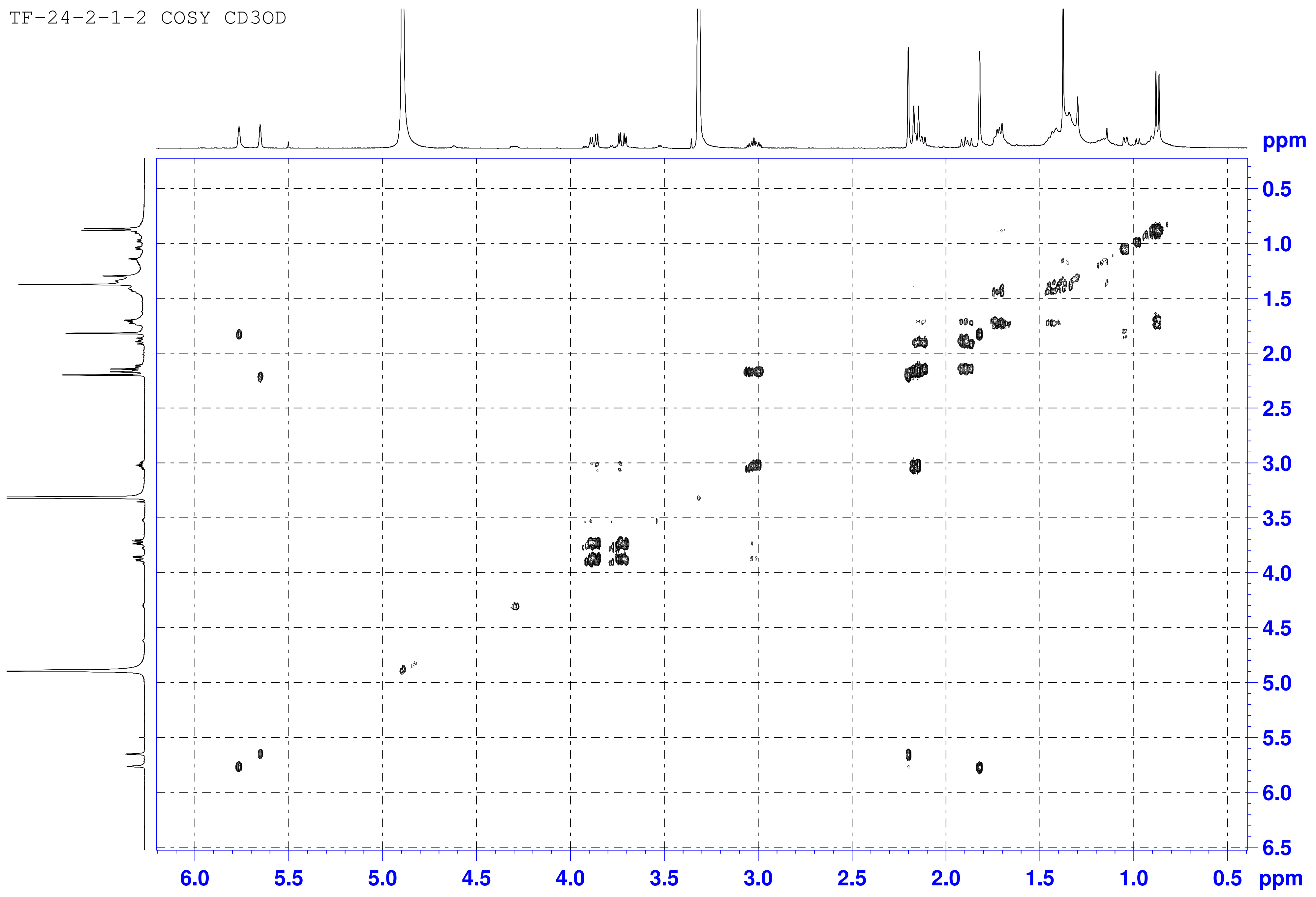

Figure S2-4 COSY Spectrum of **2** in CD_3OD

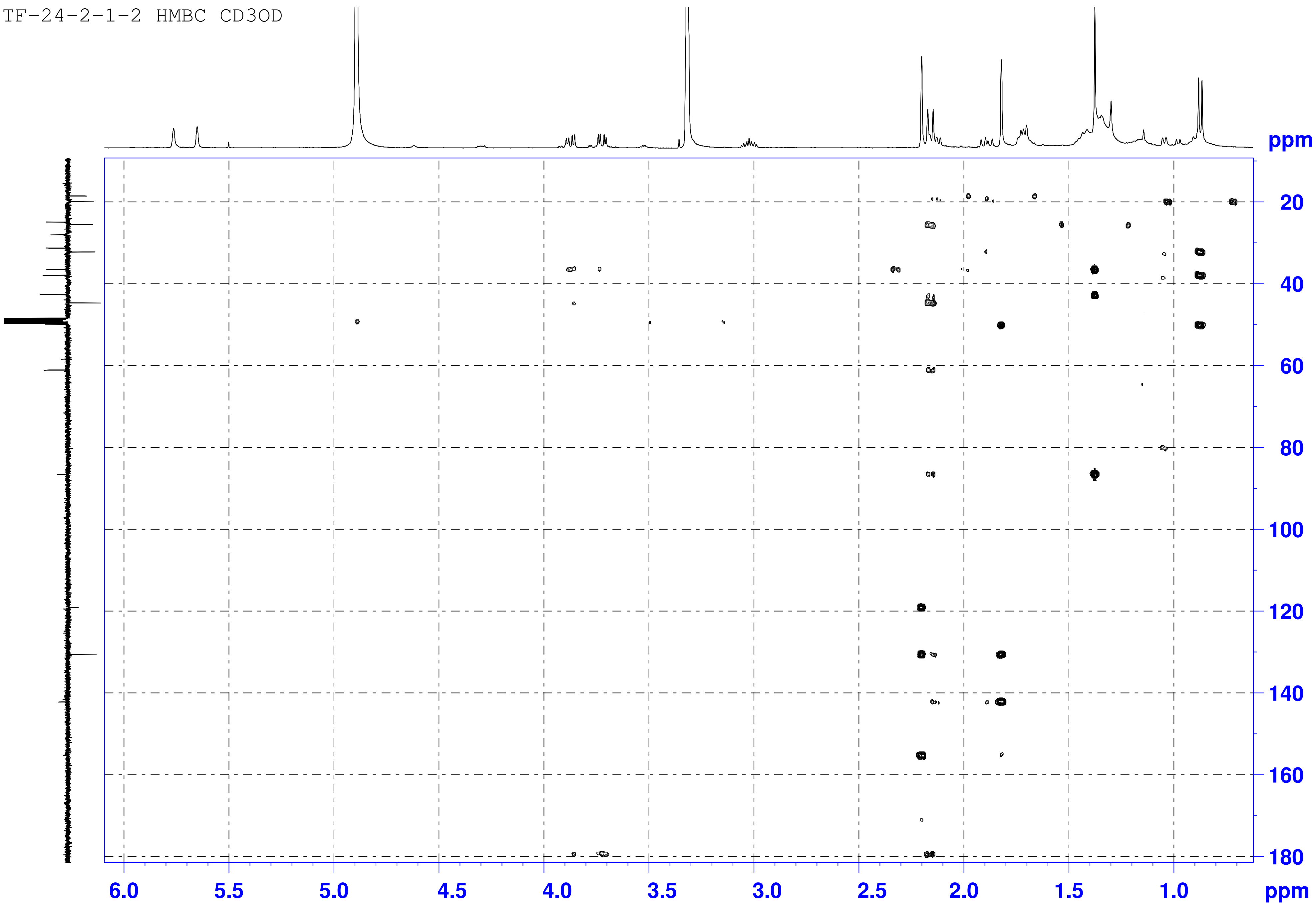

Figure S2-5 HMBC Spectrum of **2** in CD_3OD

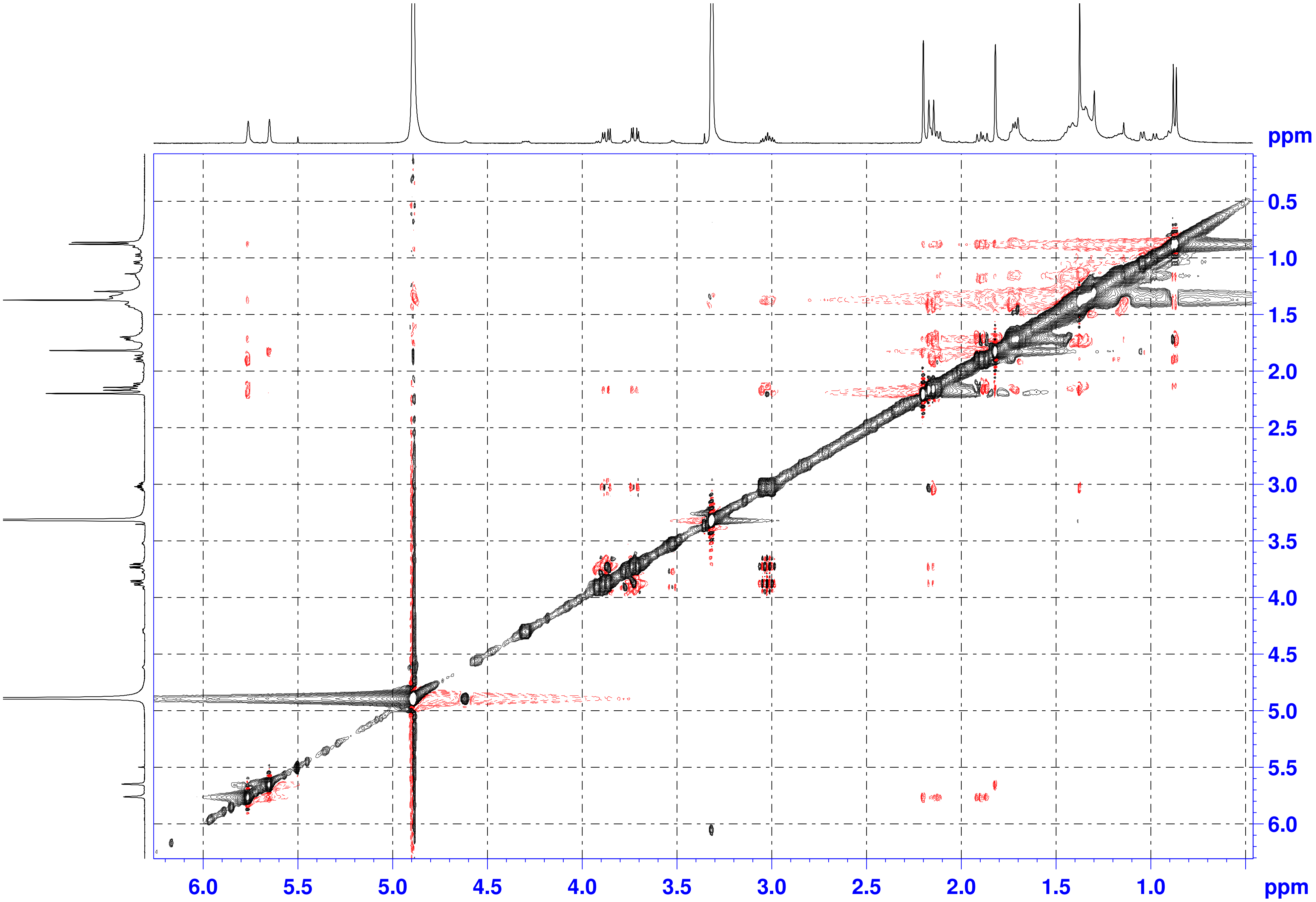

Figure S2-6 NOESY Spectrum of **2** in CD_3OD

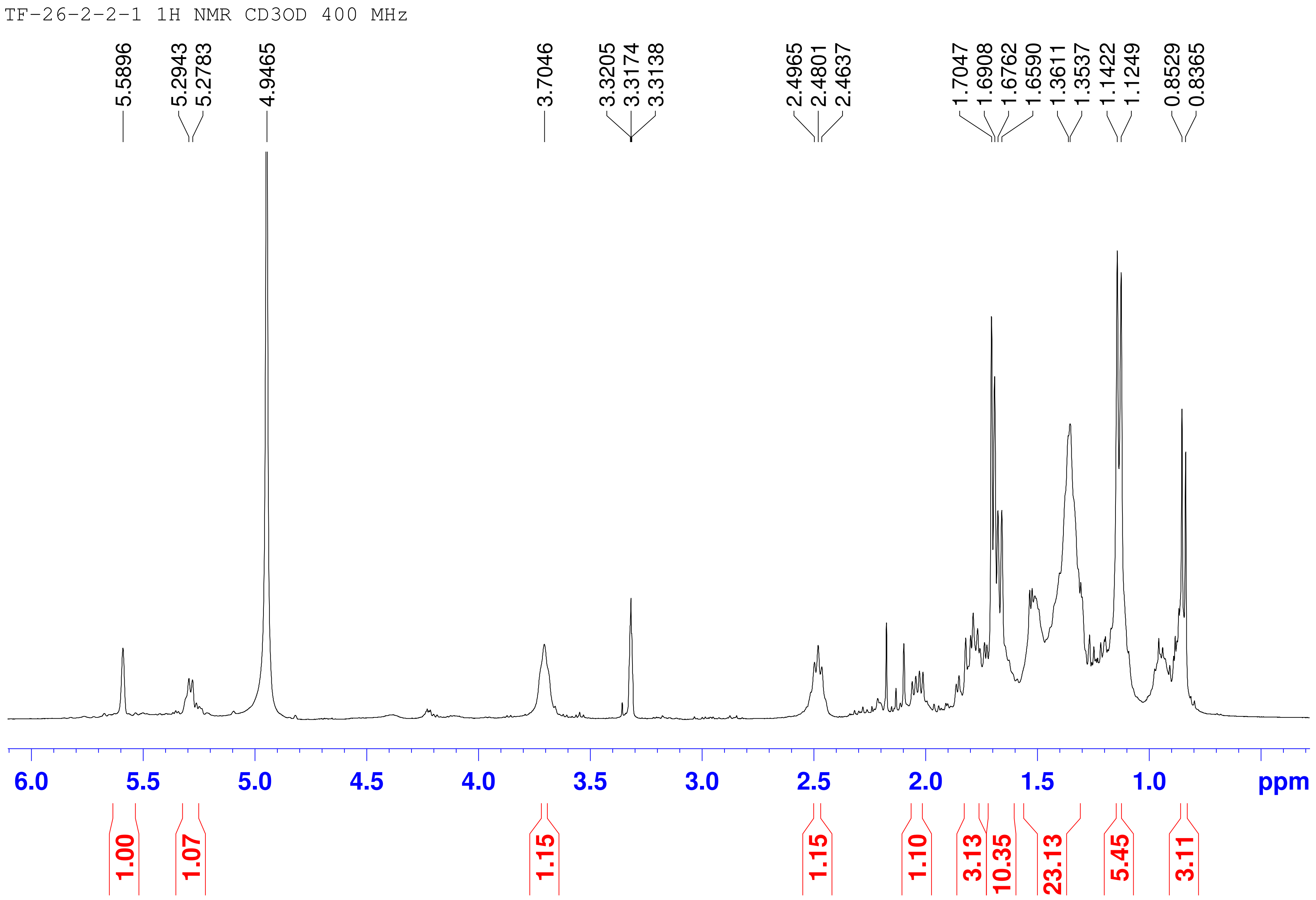

Figure S3-1 ^{1}H NMR Spectrum of **3** in CD_3OD (400 MHz)

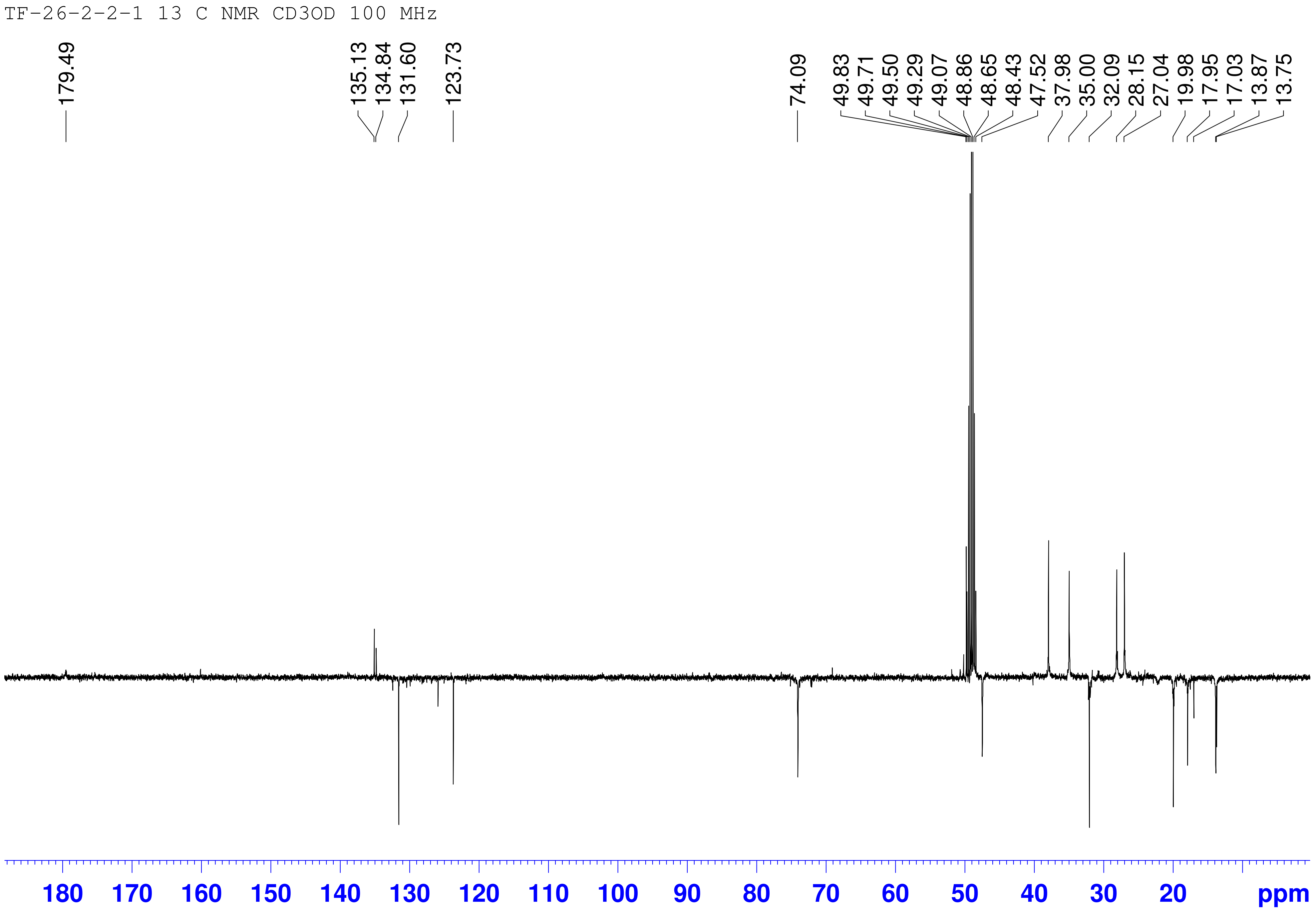

Figure S3-2 ^{13}C Spectrum of **3** in CD_3OD (100 MHz)

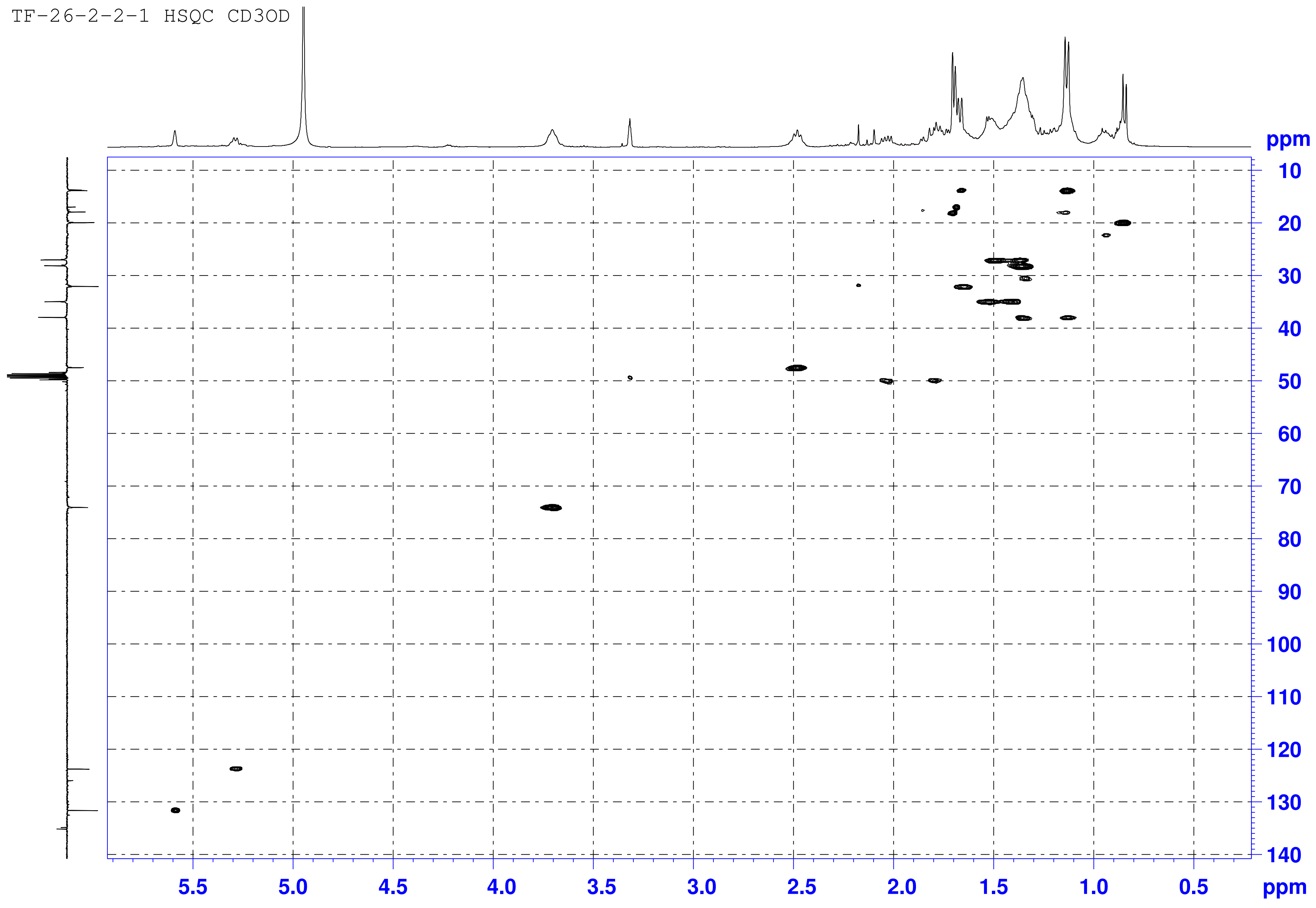

Figure S3-3 HSQC Spectrum of **3** in CD_3OD

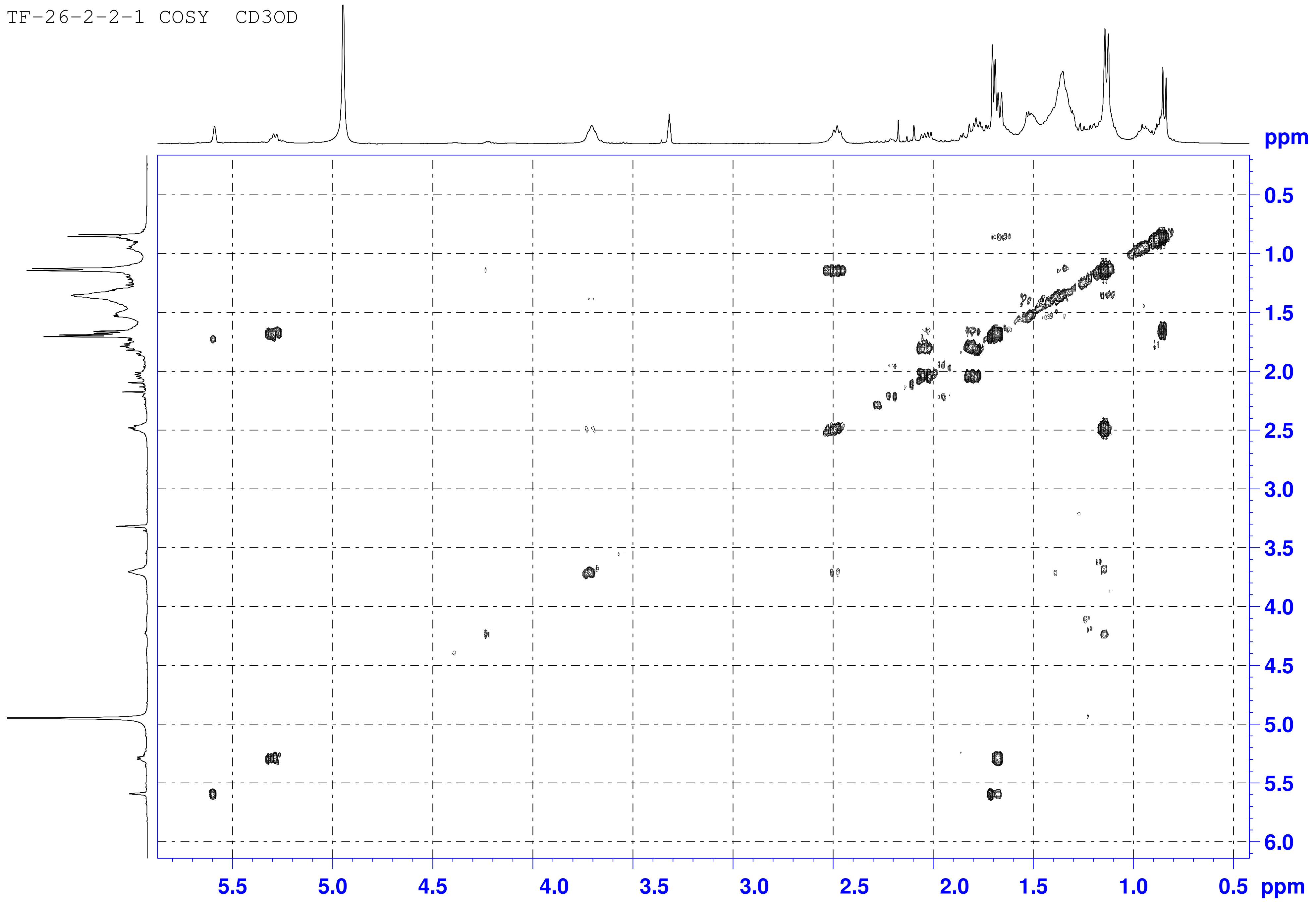

Figure S3-4 COSY Spectrum of **3** in CD_3OD

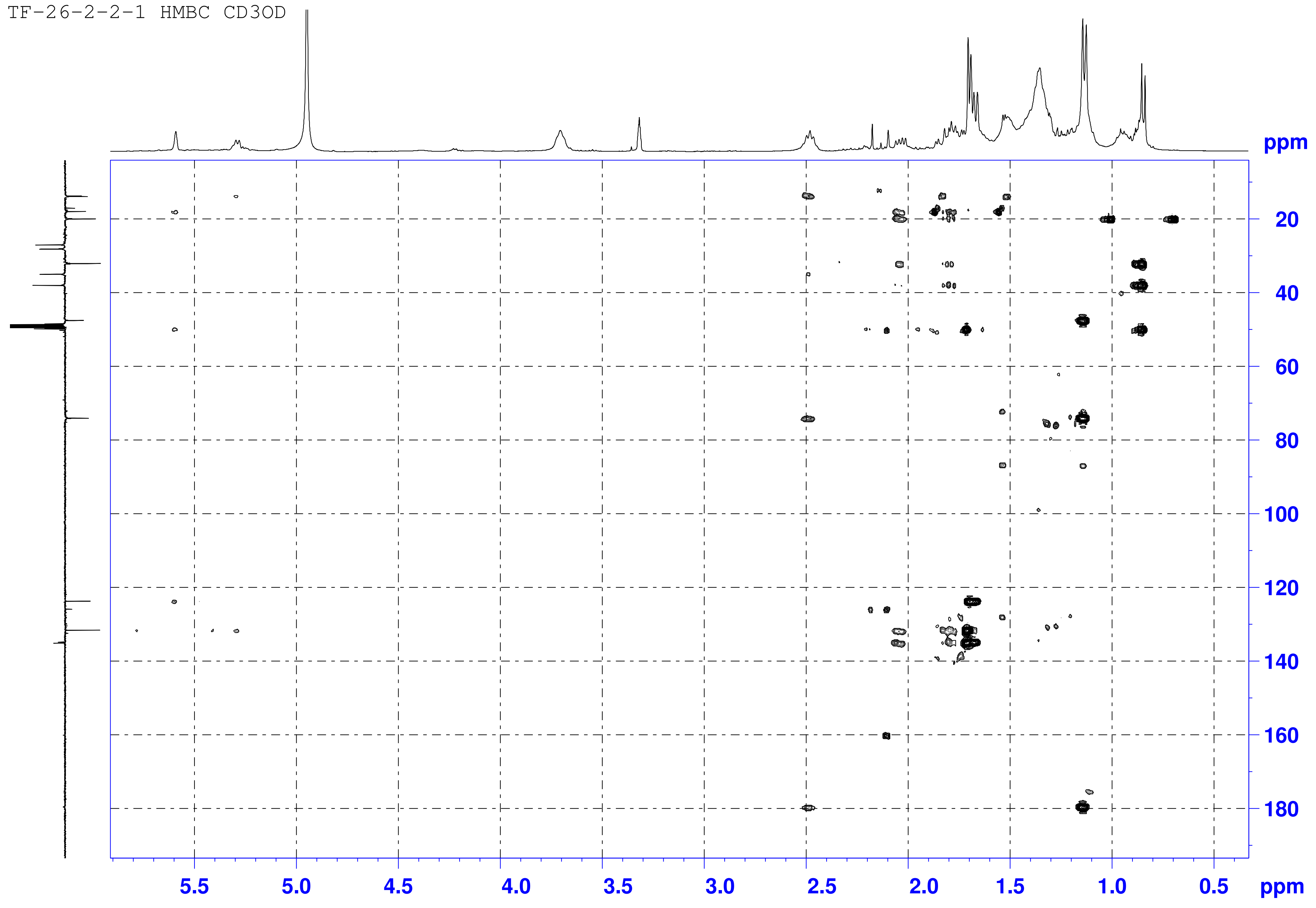

Figure S3-5 HMBC Spectrum of **3** in CD_3OD

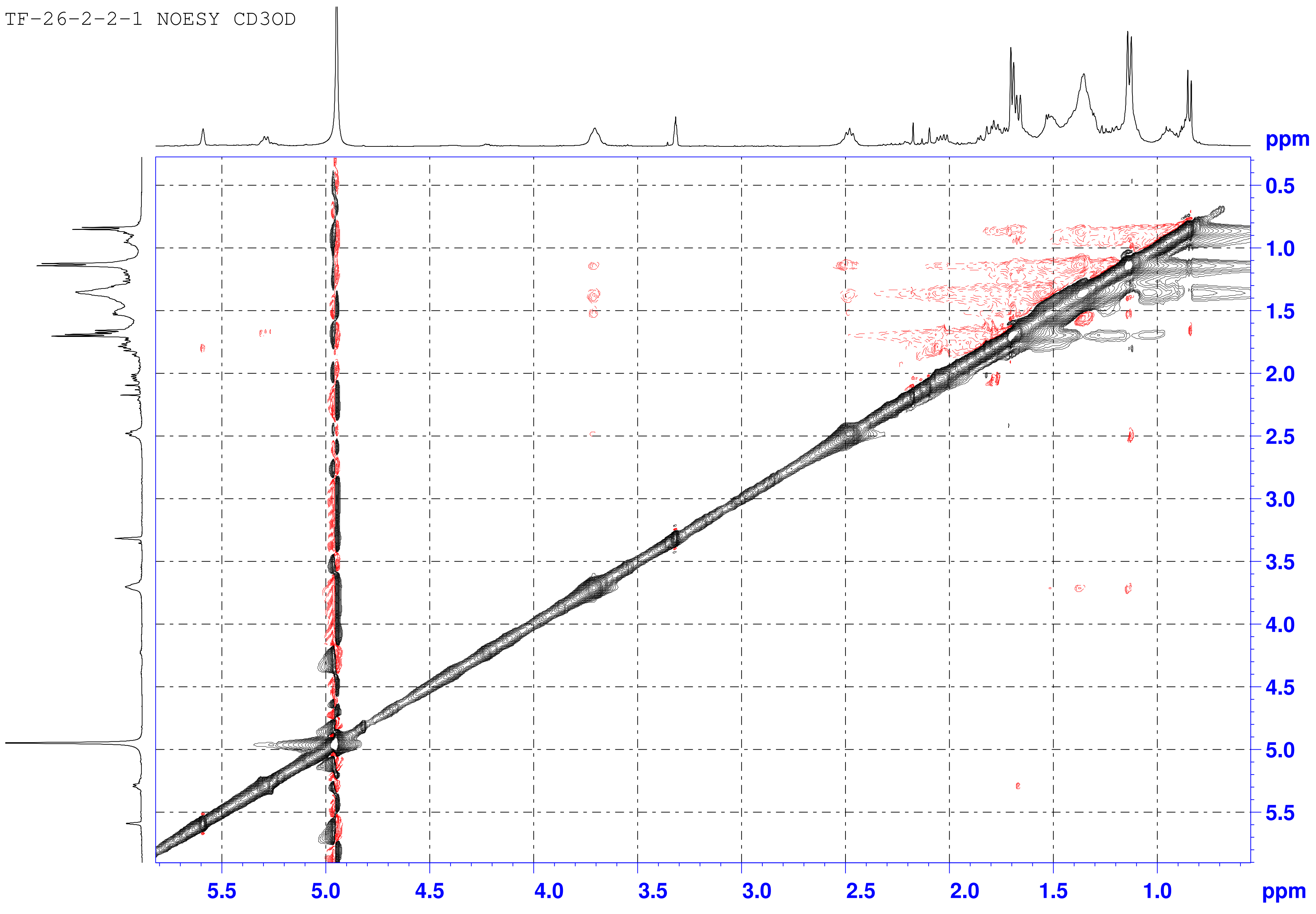

Figure S3-6 NOESY Spectrum of **3** in CD_3OD

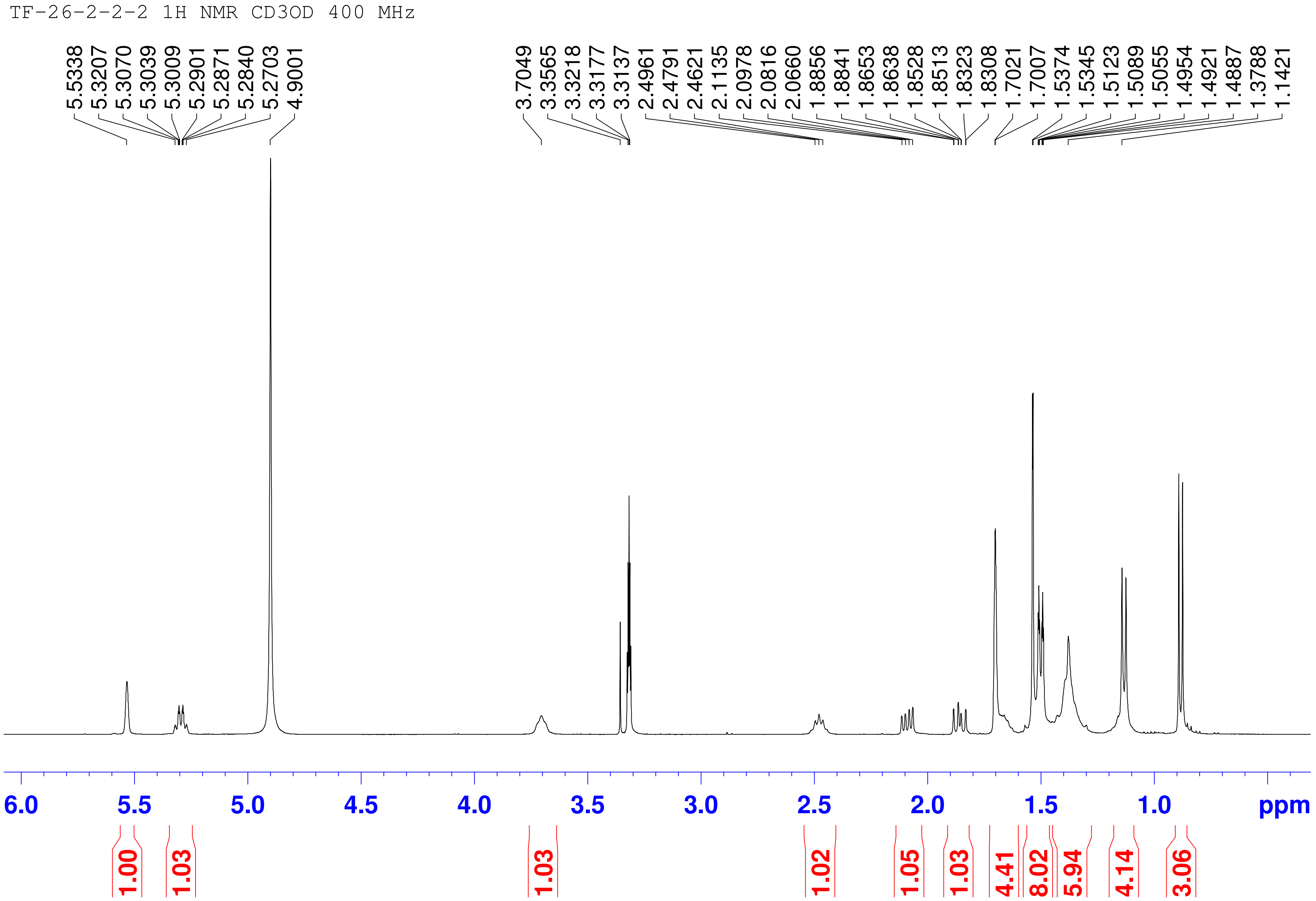

Figure S4-1 ^{1}H NMR Spectrum of **4** in CD_3OD (400 MHz)

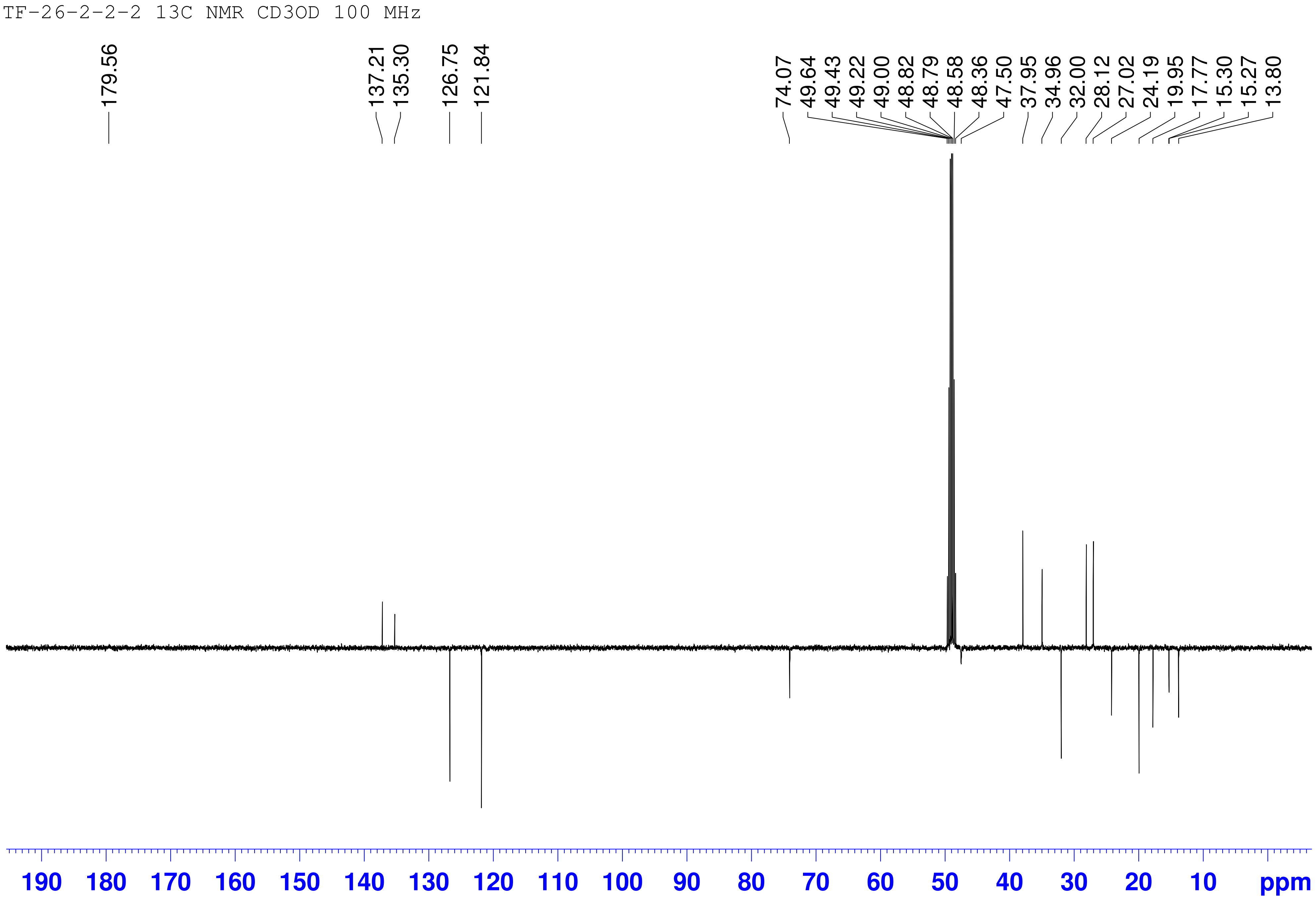

Figure S4-2 ^{13}C Spectrum of **4** in CD_3OD (100 MHz)

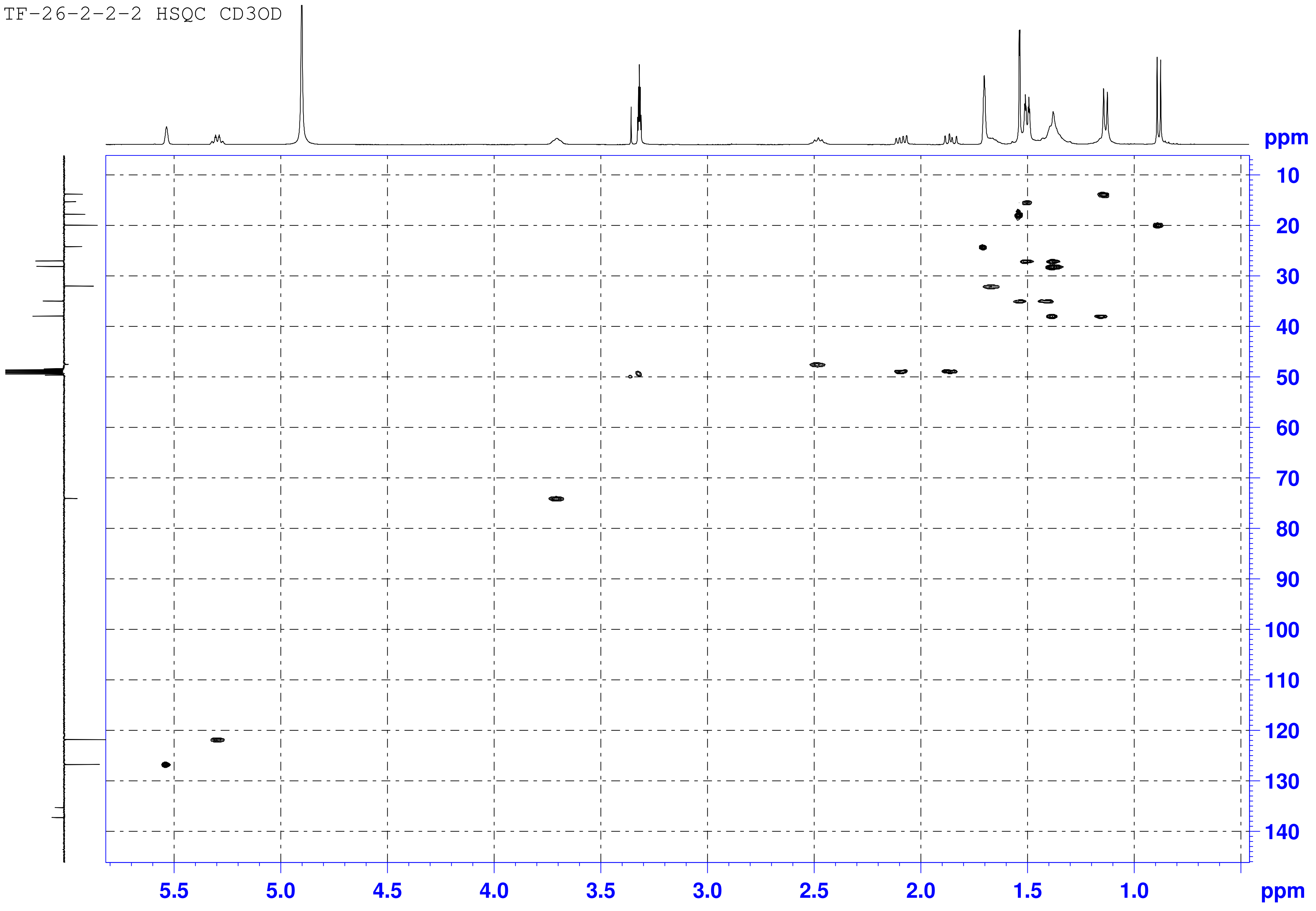

Figure S4-3 HSQC Spectrum of **4** in CD_3OD

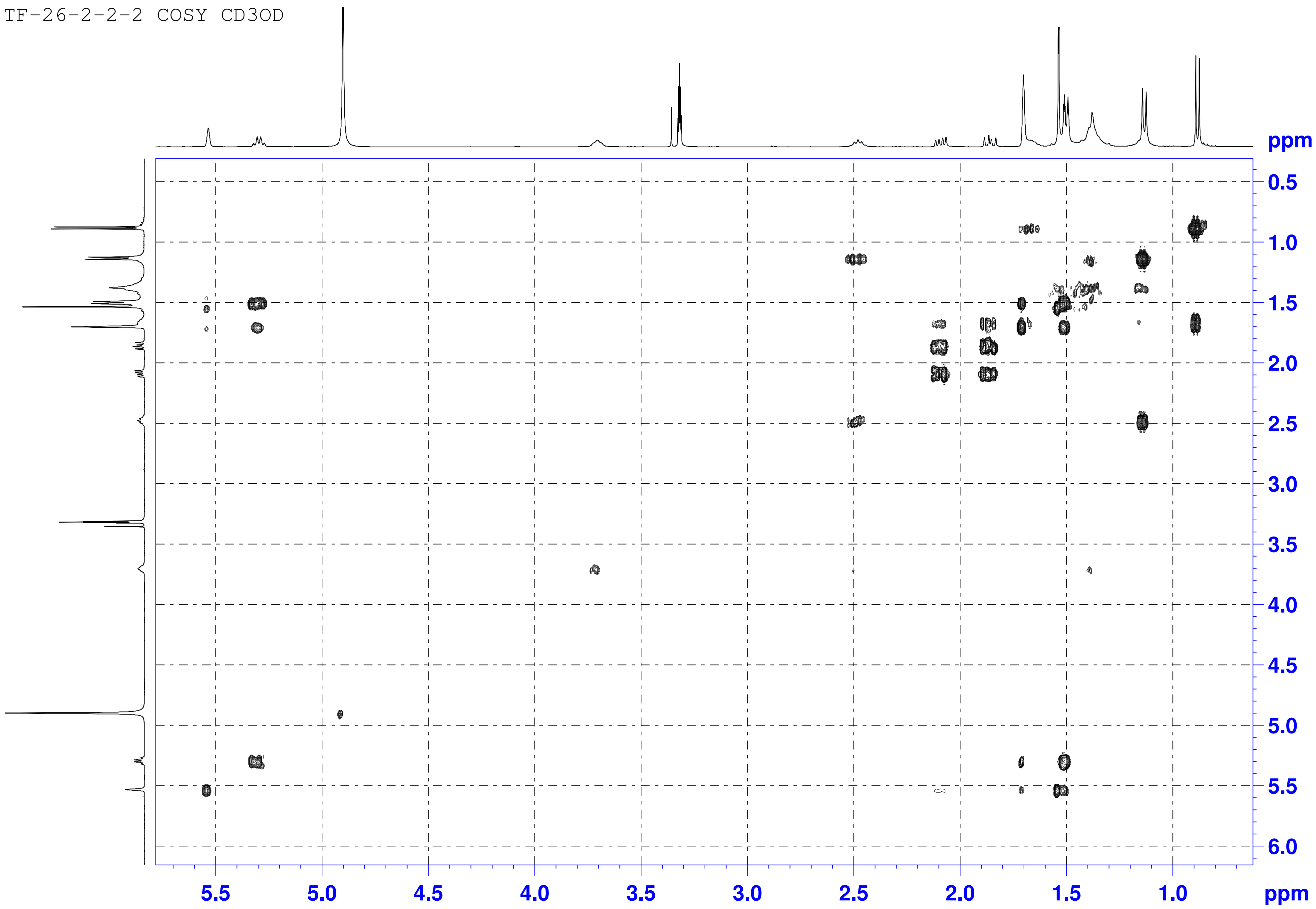

Figure S4-4 COSY Spectrum of **4** in CD_3OD

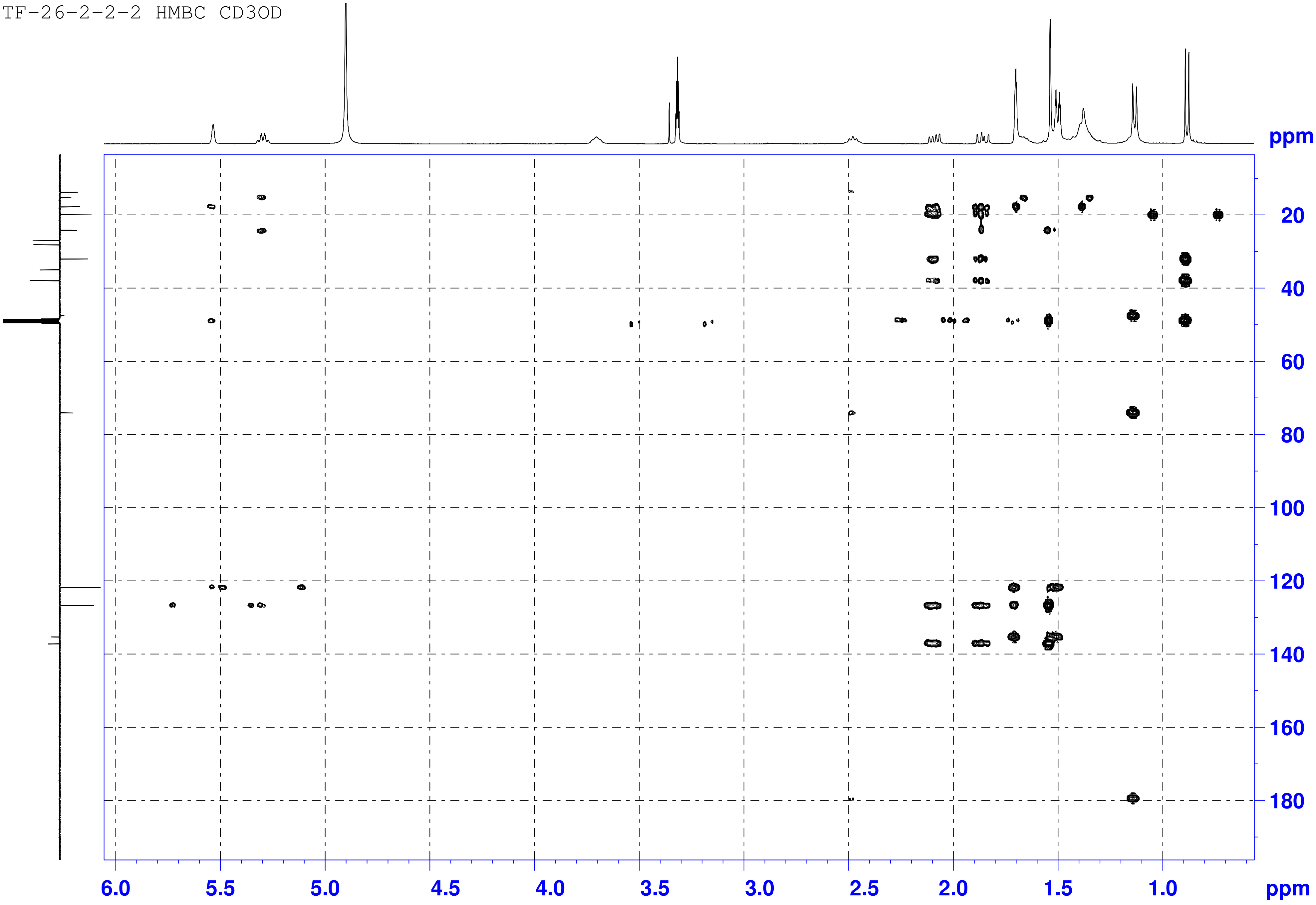

Figure S4-5 HMBC Spectrum of **4** in CD_3OD

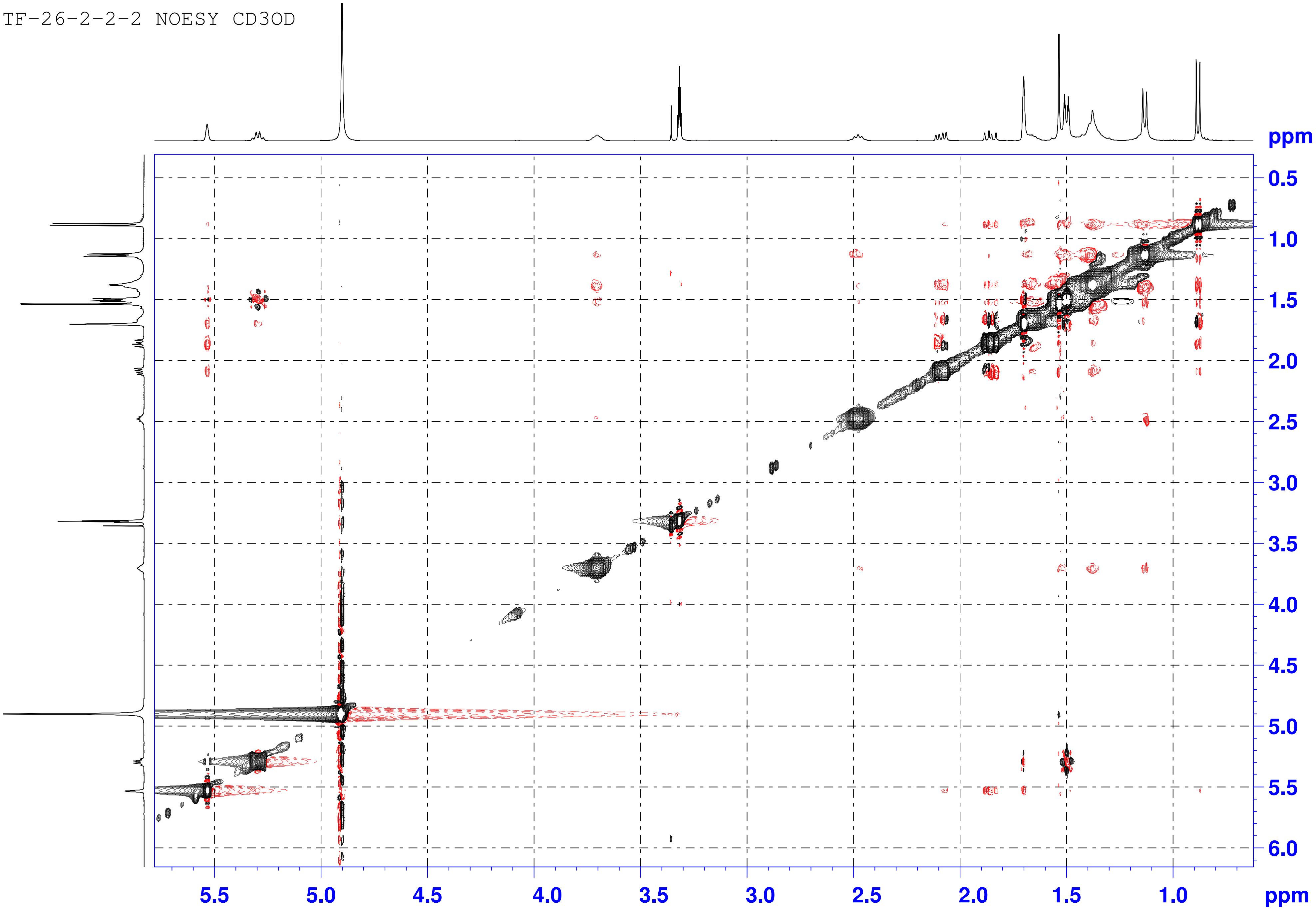

Figure S4-6 NOESY Spectrum of **4** in CD_3OD

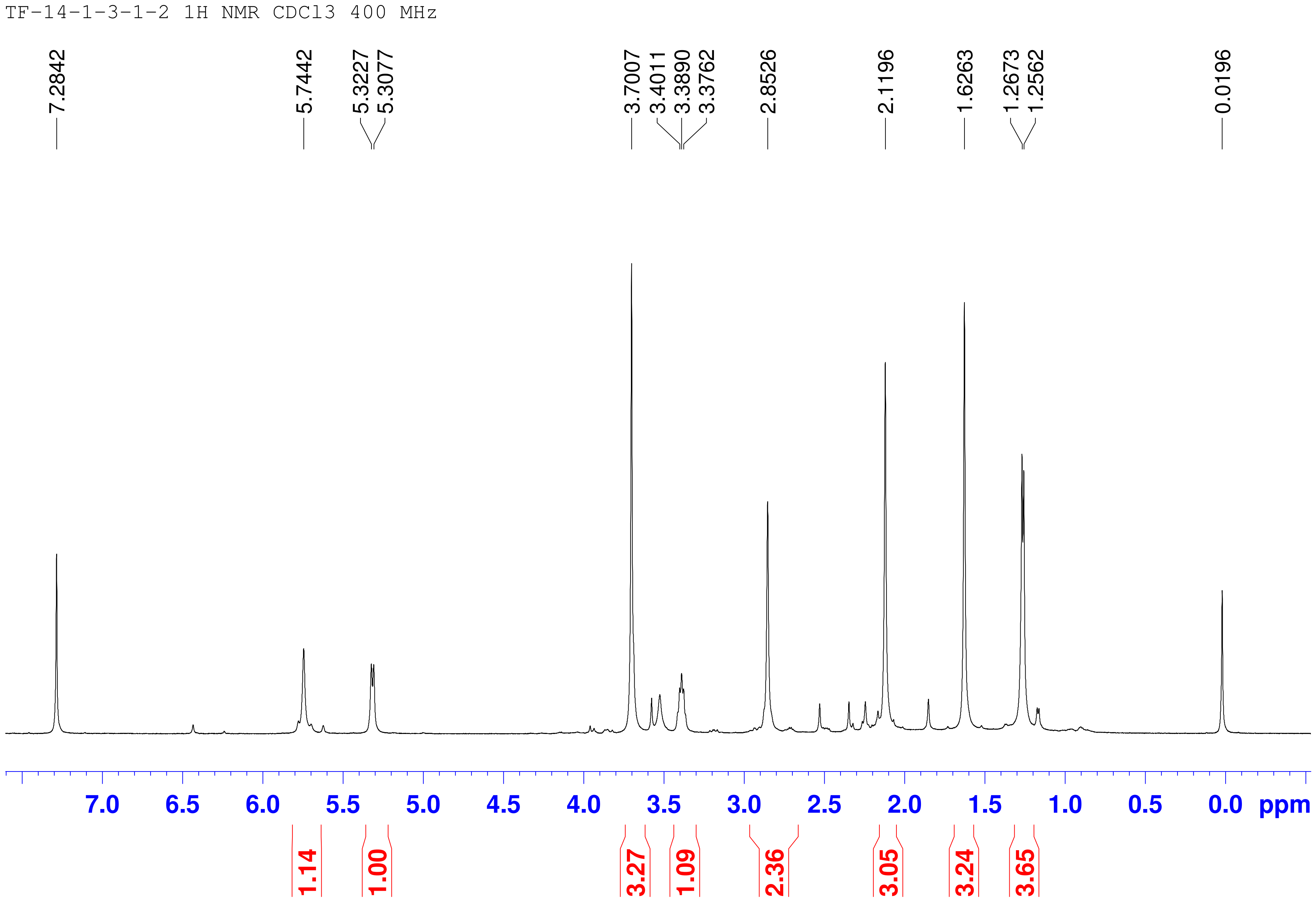

Figure S5-1 ^{1}H NMR Spectrum of **5** in $CDCl_3$ (400 MHz)

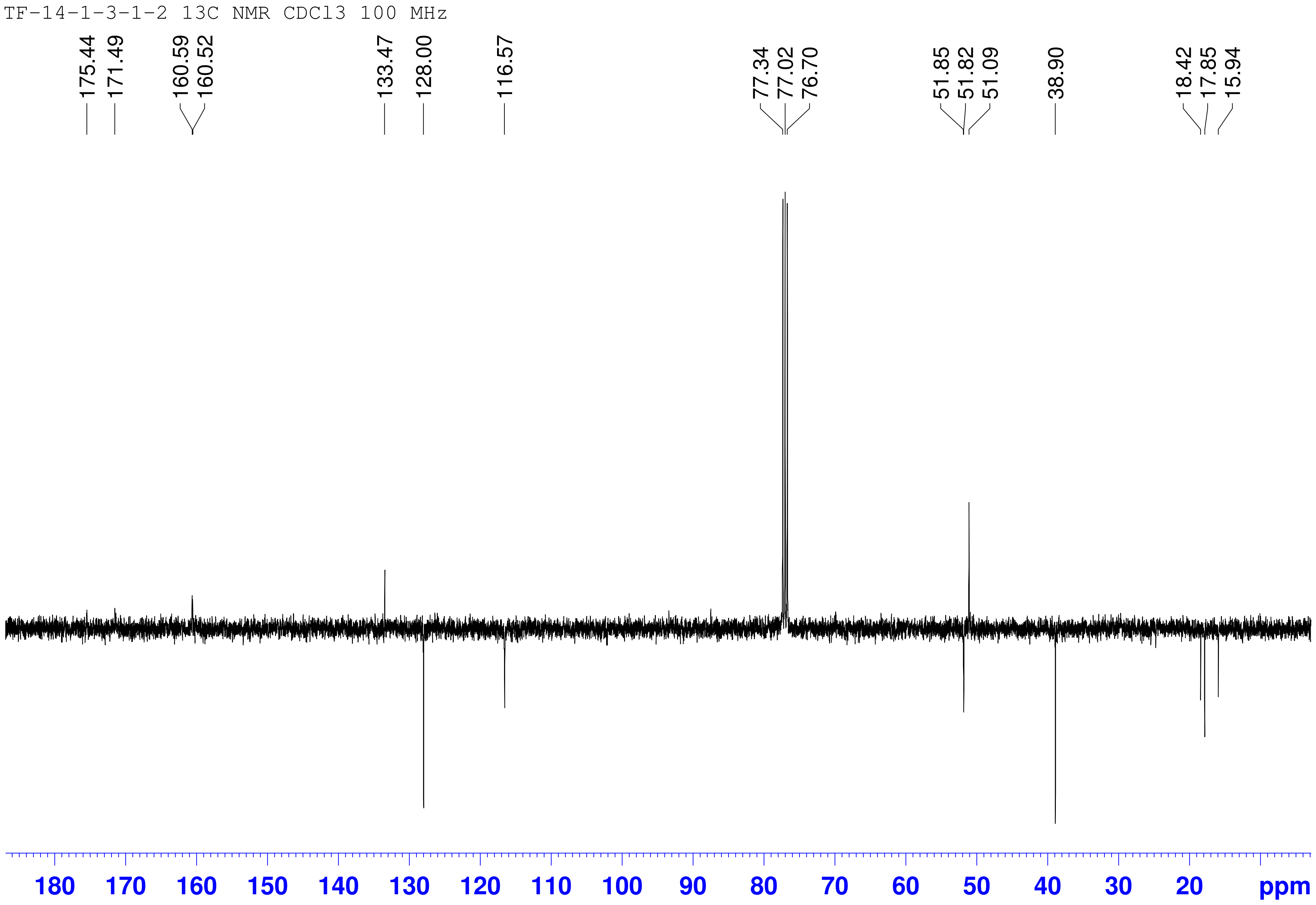

Figure S5-2 ^{13}C Spectrum of **5** in $CDCl_3$ (100 MHz)

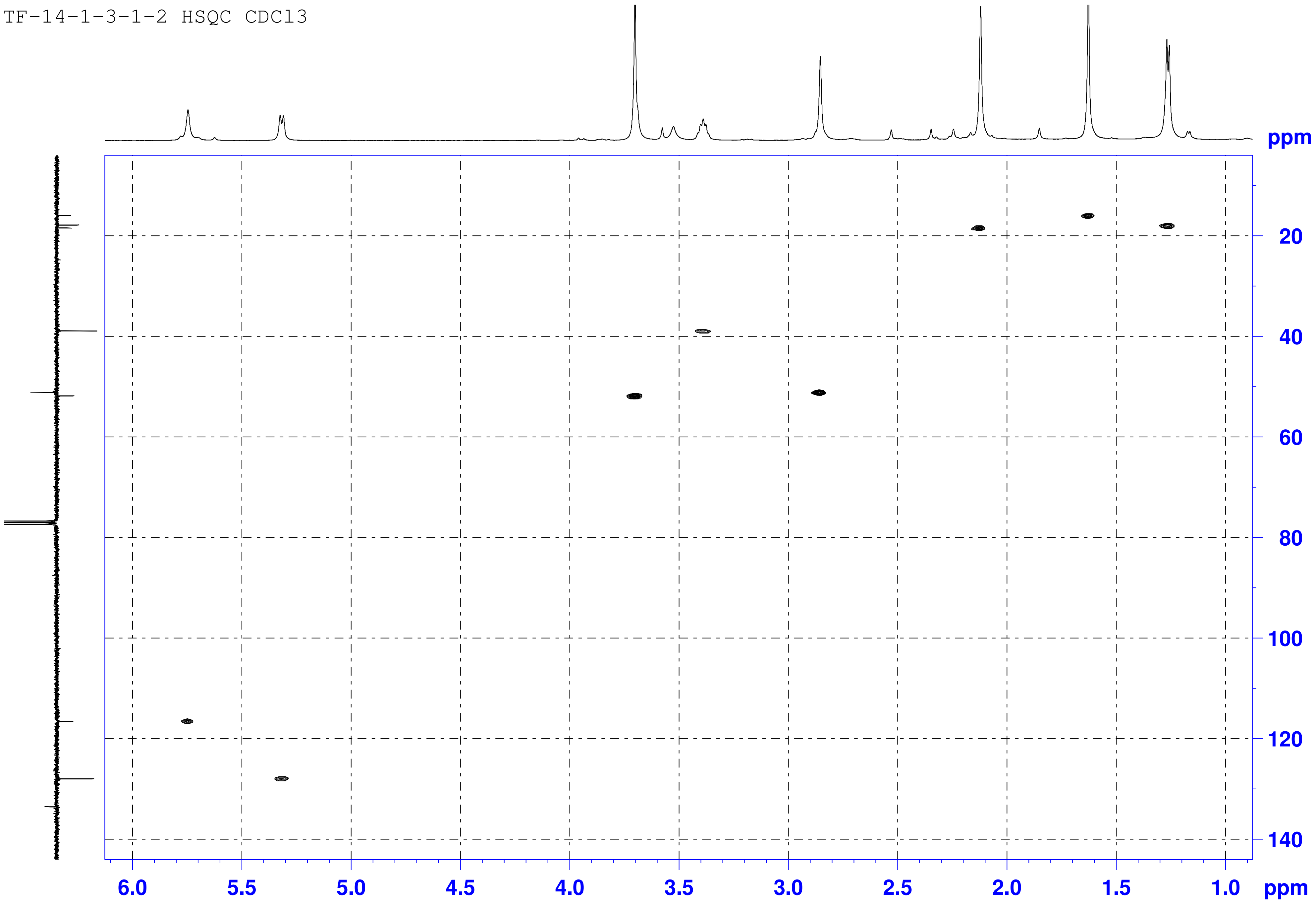

Figure S5-3 HSQC Spectrum of **5** in $CDCl_3$

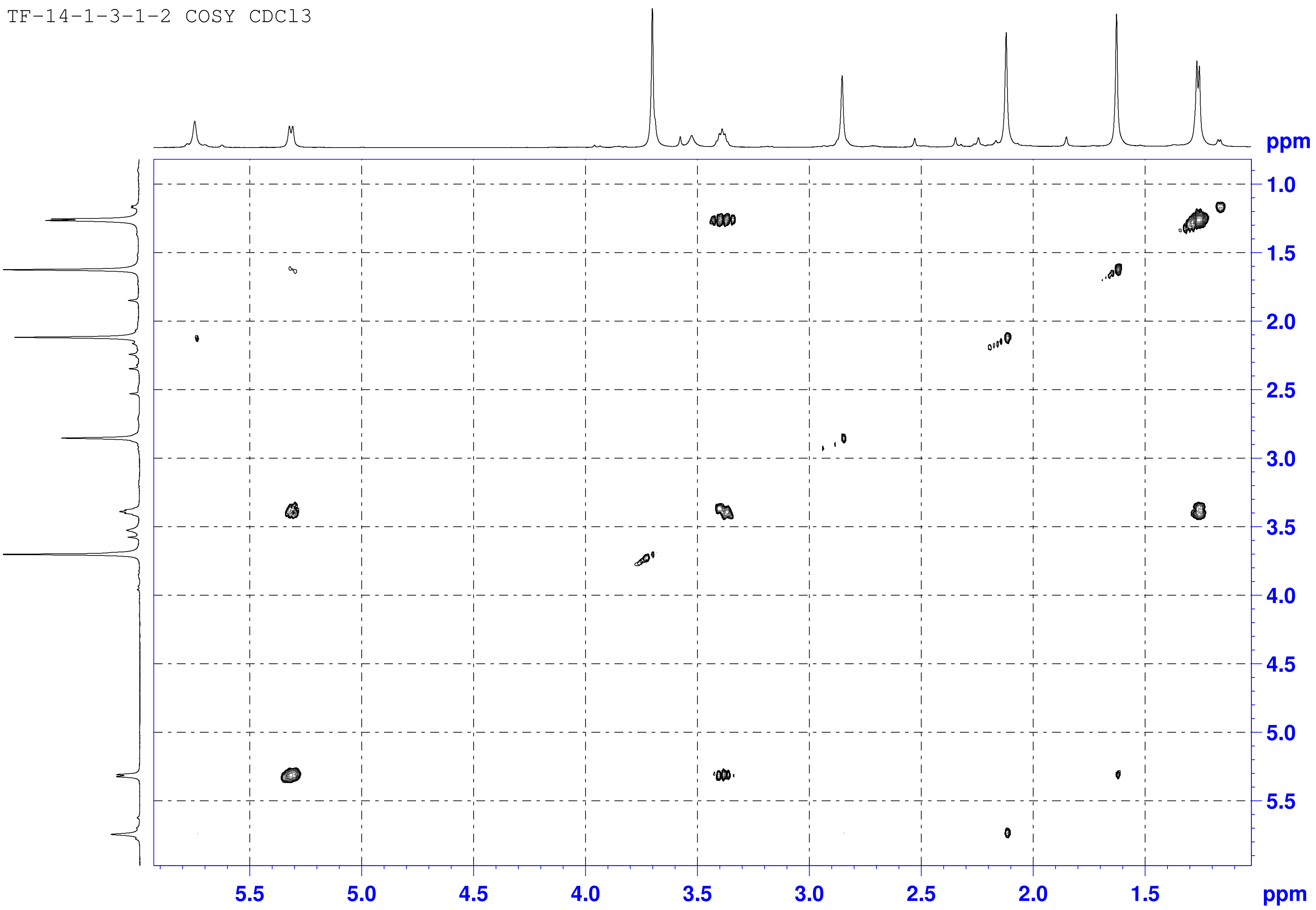

Figure S5-4 COSY Spectrum of **5** in $CDCl_3$

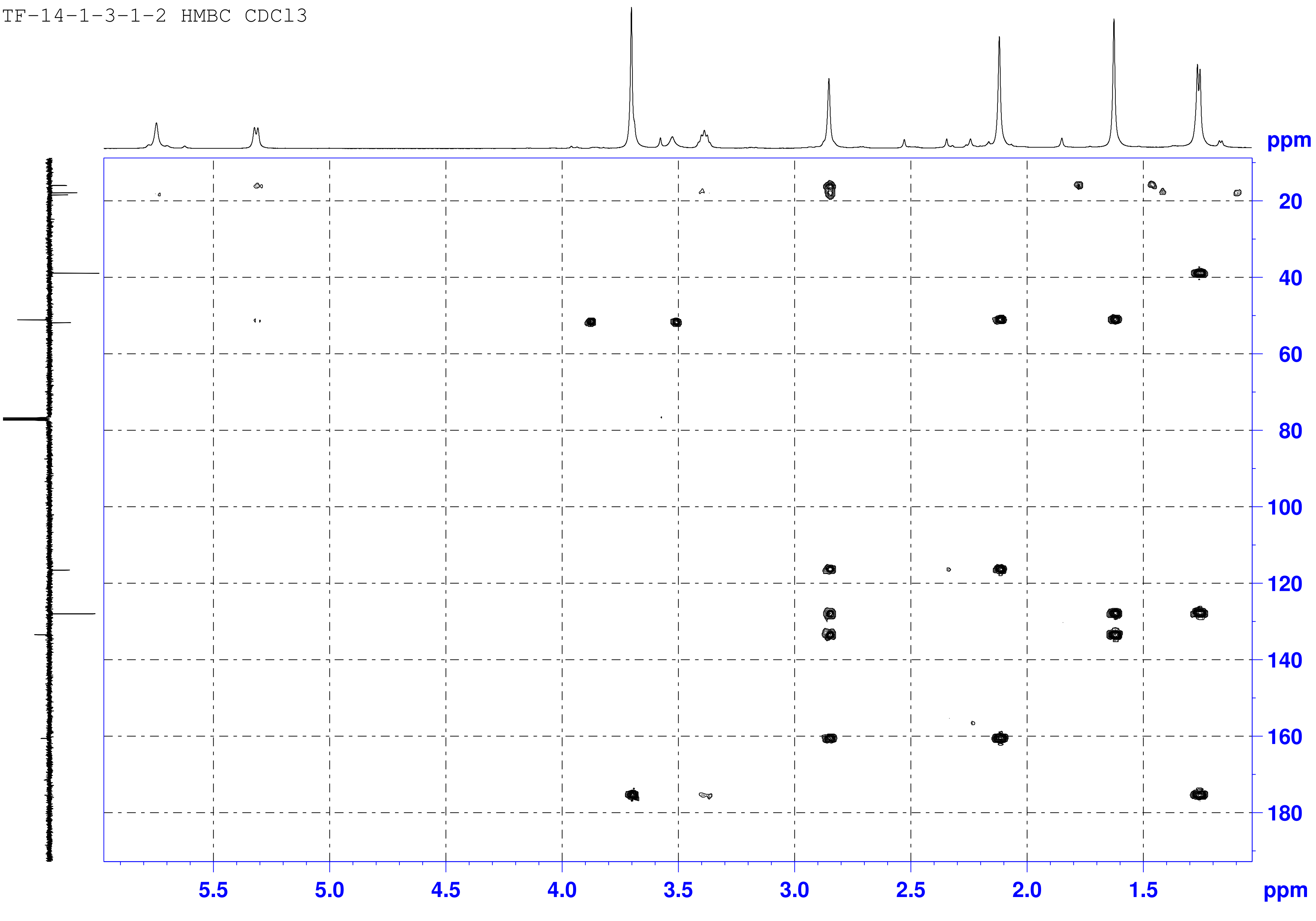

Figure S5-5 HMBC Spectrum of **5** in $CDCl_3$

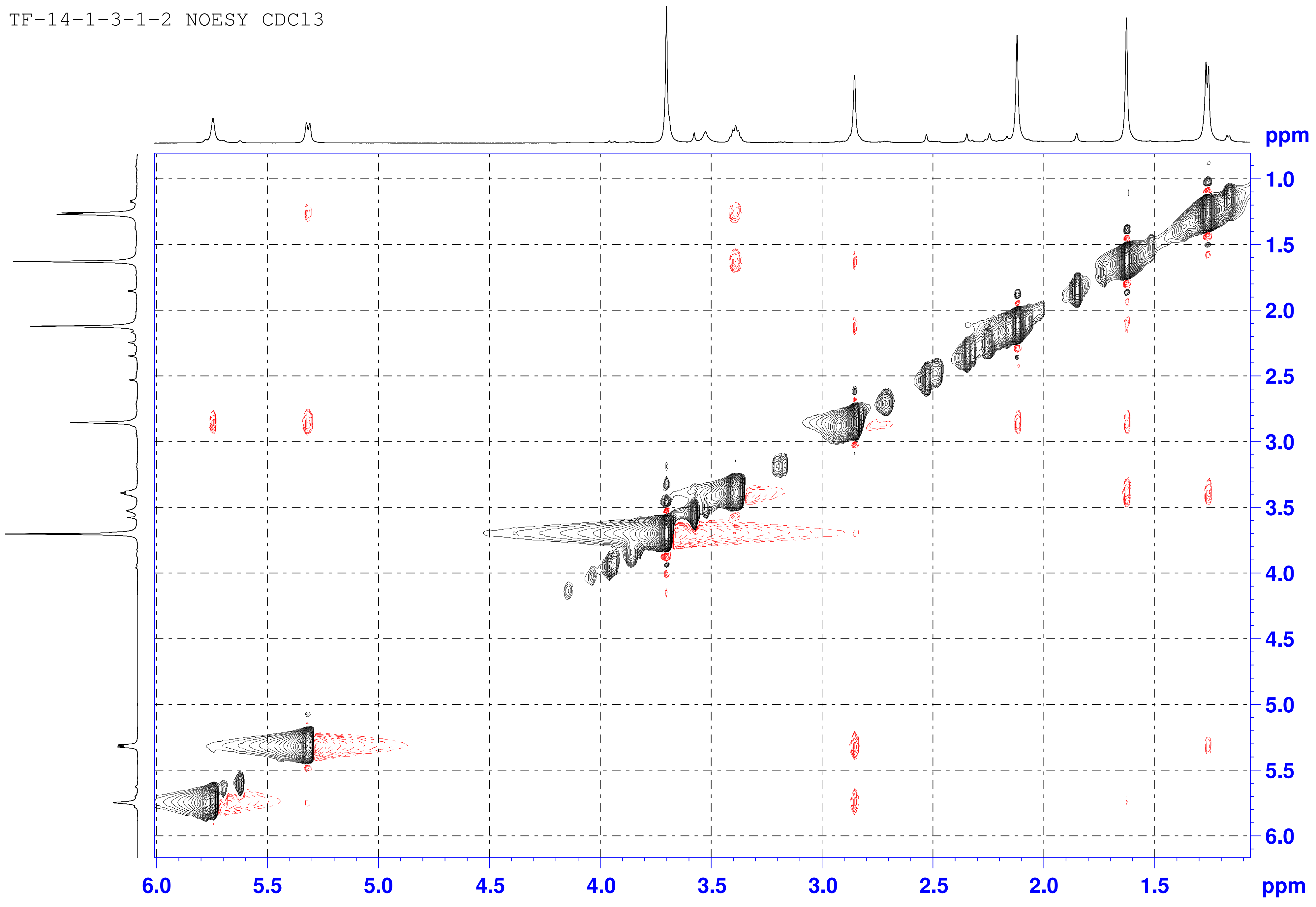

Figure S5-6 NOESY Spectrum of **5** in $CDCl_3$

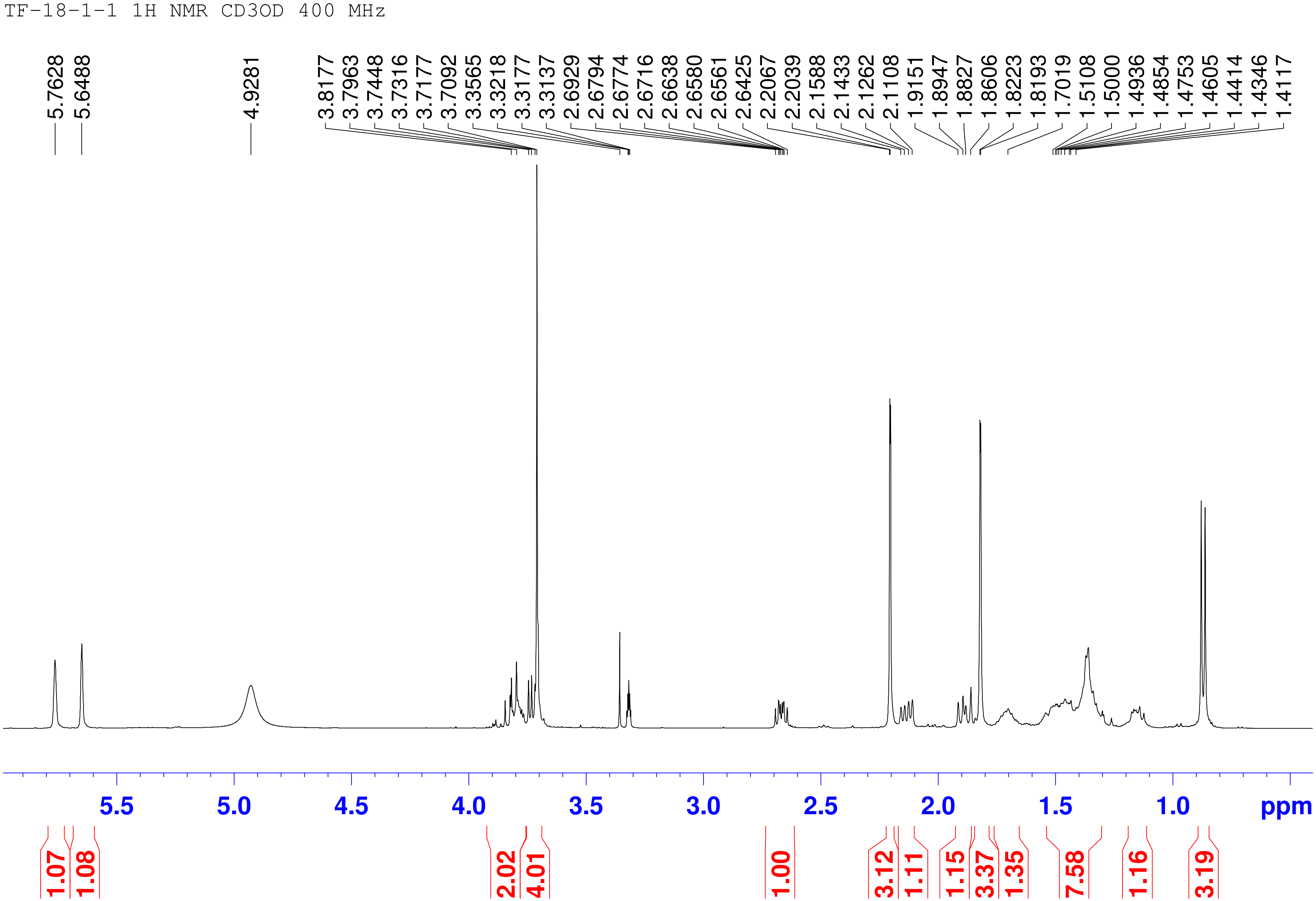

Figure S6-1 ^{1}H NMR Spectrum of **6** in CD_3OD (400 MHz)

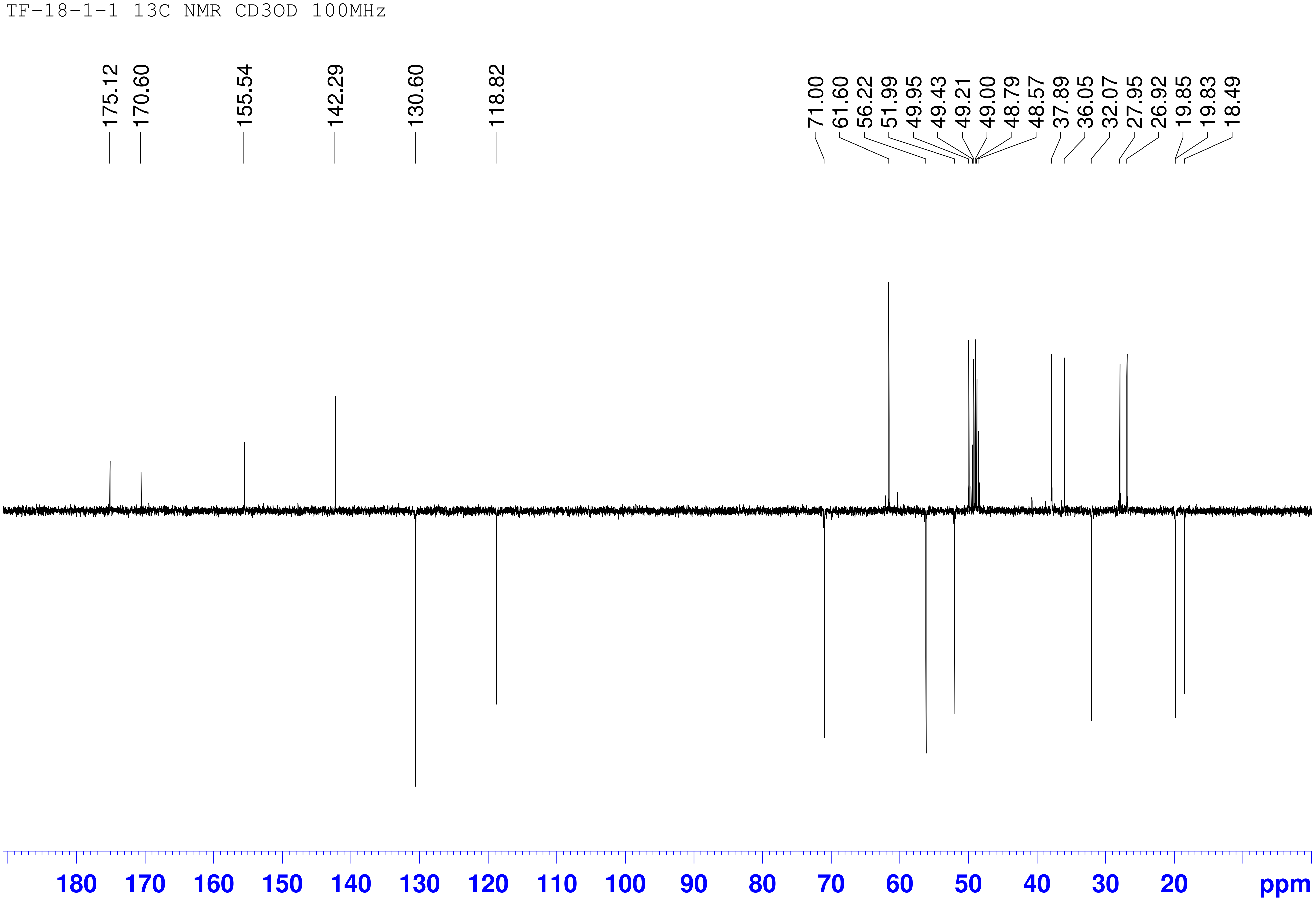

Figure S6-2 ^{13}C NMR Spectrum of **6** in CD_3OD (100 MHz)

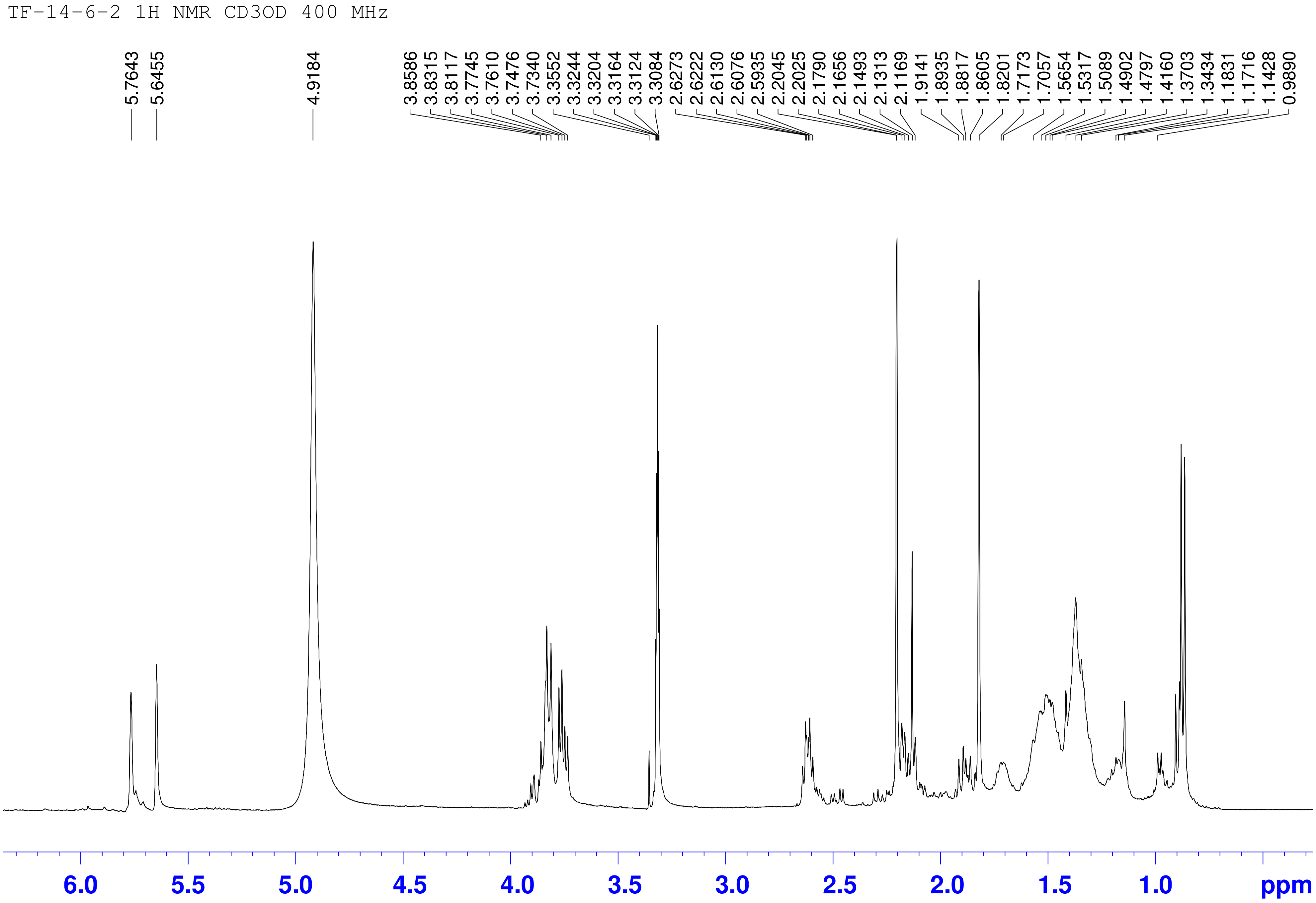

Figure S7-1 ^{1}H NMR Spectrum of **7** in CD_3OD (400 MHz)

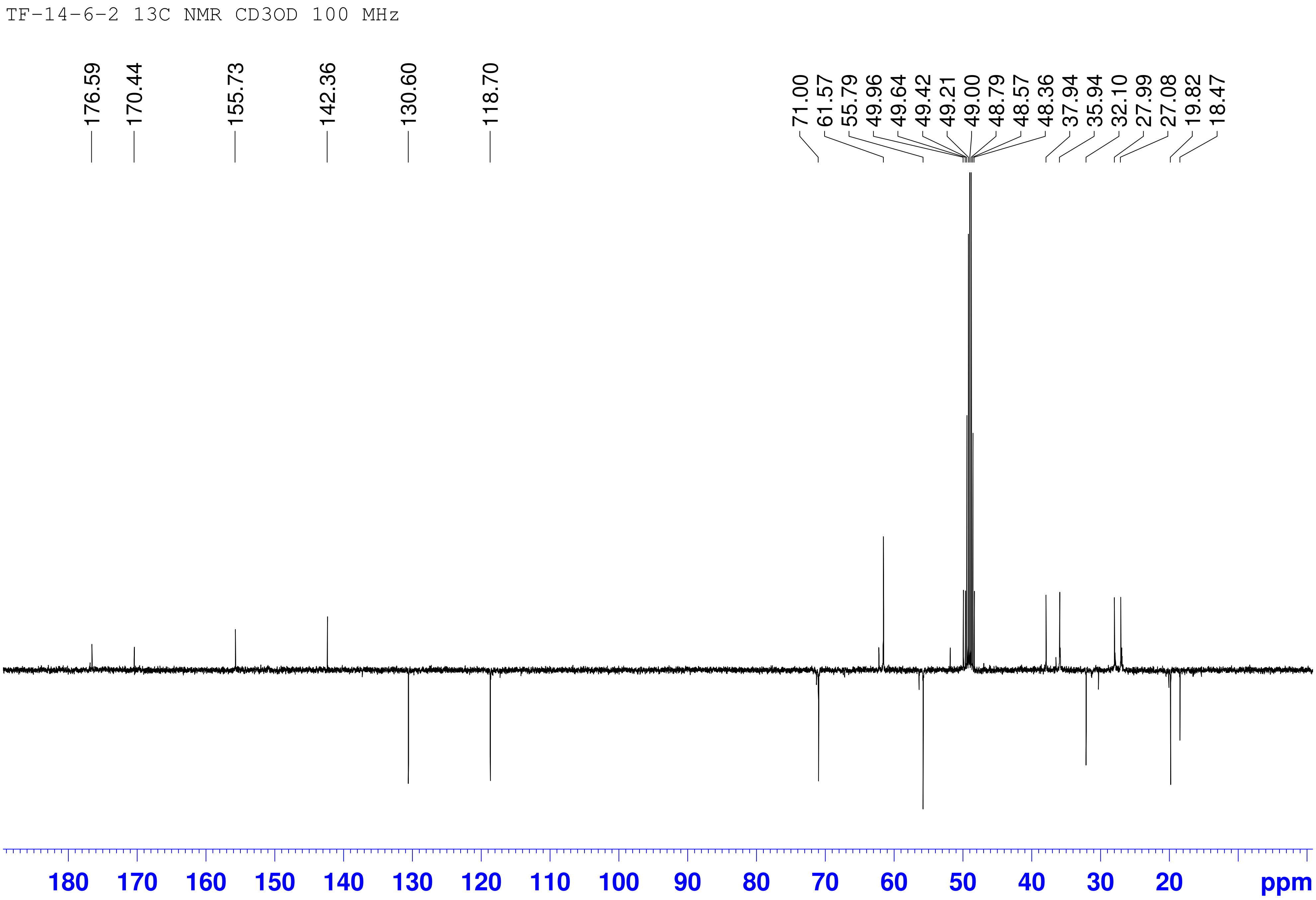

Figure S7-2 ^{13}C NMR Spectrum of **7** in CD_3OD (100 MHz)

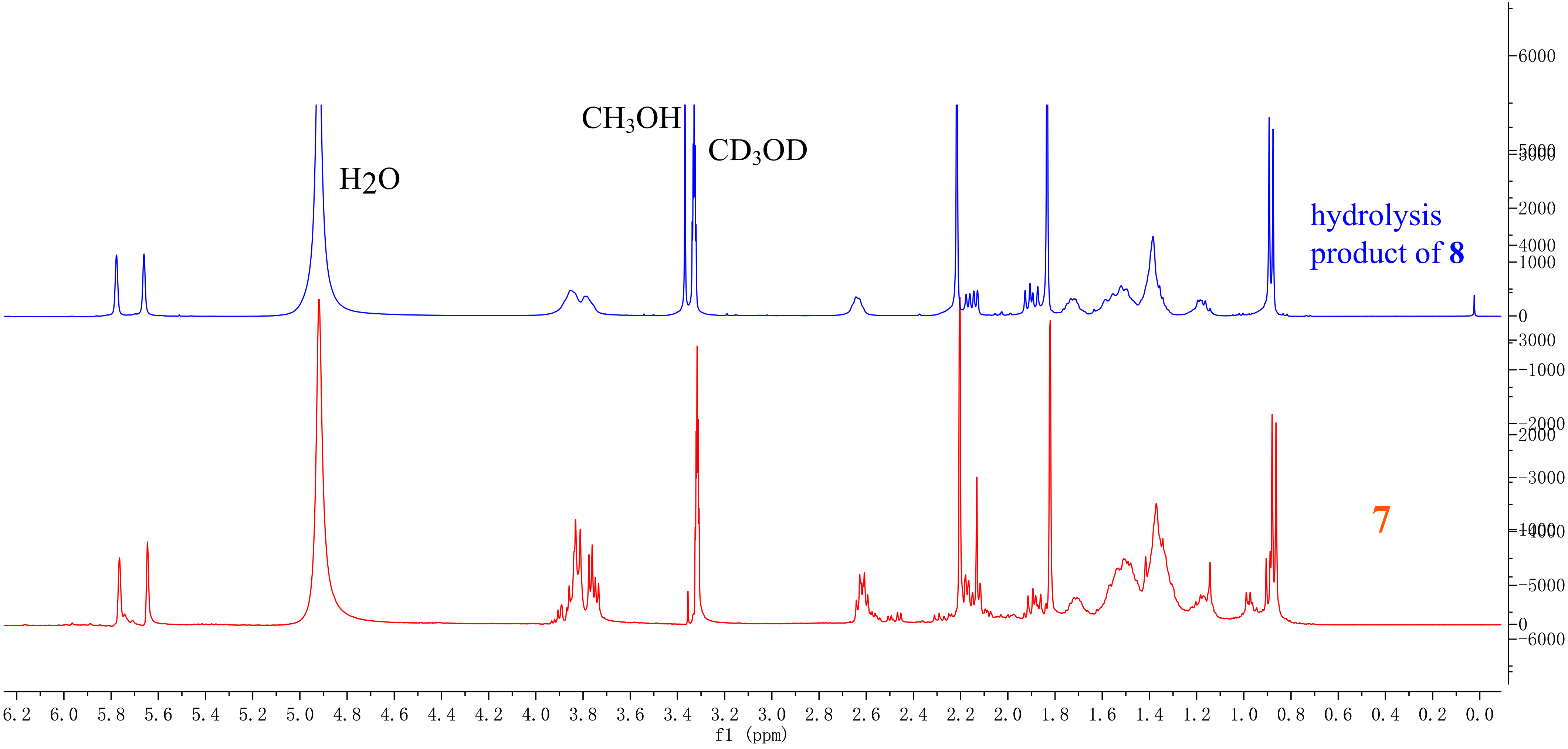

Figure S8-1 Comparison of ^{1}H NMR Spectra of **7** and hydrolysis product of **8** (CD_3OD, 400 MHz)

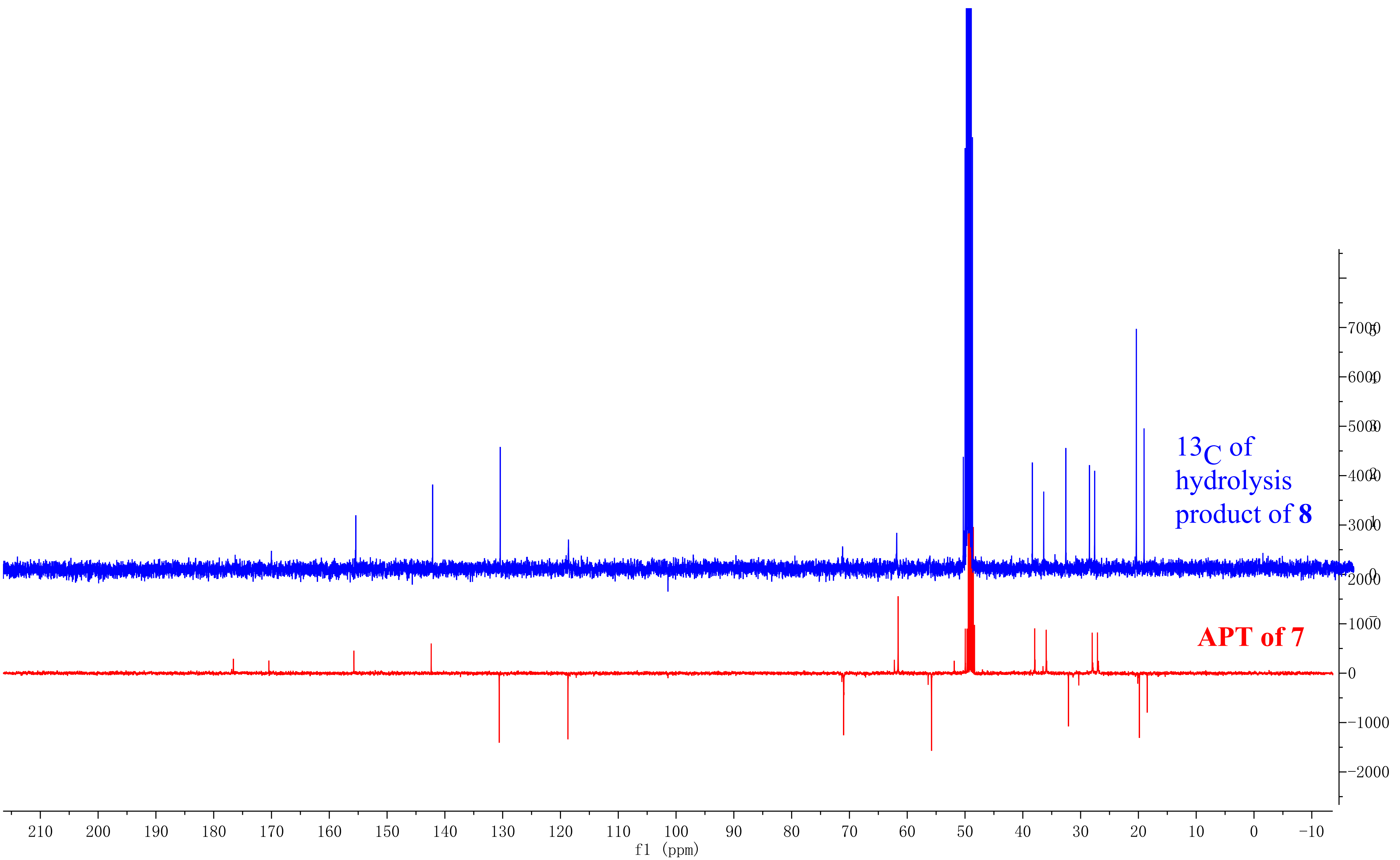

Figure 8-2 Comparison of ^{13}C NMR Spectra of **7** and hydrolysis product of **8** (CD_3OD, 100 MHz)

Table S1. ^{1}H (400 MHz) and ^{13}C (100 MHz) NMR Data for 7 in CD_3OD

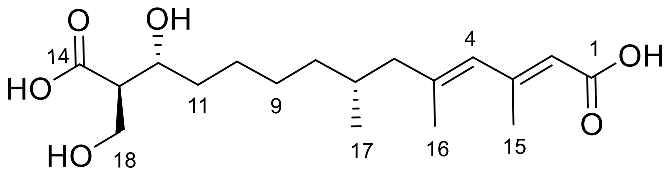

7/8a

Position	δ_H	δ_C
1		170.4, C
2	5.65, br s	118.7, CH
3		155.7, C
4	5.76, br s	130.6, CH
5		142.4, C
6	2.14, m; 1.89, m	50.1, CH_2
7	1.71, m	32.1, CH
8	1.35, m; 1.17, m	37.9, CH_2
9	1.49, m; 1.37, m	27.1, CH_2
10	1.37, m	28.0, CH_2
11	1.52, m	35.9, CH_2
12	3.83, m	71.0, CH
13	2.62, m	55.8. CH
14		176.6, C
15	2.20, d (0.9)	19.8, CH_3
16	1.82, s	18.5, CH_3
17	0.87, d (6.6)	19.8, CH_3
18	3.84, overlapped	61.6, CH_2
	3.75, dd (10.8, 5.5)	
